# Advancements in Biodegradable Active Films for Food Packaging: Effects of Nano/Microcapsule Incorporation

**DOI:** 10.3390/foods11050760

**Published:** 2022-03-06

**Authors:** Fatemeh Baghi, Adem Gharsallaoui, Emilie Dumas, Sami Ghnimi

**Affiliations:** 1 Laboratoire d’Automatique, de Génie des Procédés et de Génie Pharmaceutique, CNRS, University Claude Bernard Lyon 1, 43 Bd 11 Novembre 1918, 69622 Villeurbanne, France; fbaghi@isara.fr (F.B.); adem.gharsallaoui@univ-lyon1.fr (A.G.); emilie.dumas@univ-lyon1.fr (E.D.); 2 Institut Supérieur d’Agriculture et Agroalimentaire Rhône-Alpes (ISARA), 23 Rue Jean Baldassini, CEDEX 07, 69364 Lyon, France

**Keywords:** biodegradable packaging, active packaging, antimicrobial agent, antioxidant agent, nano/microencapsulation, biopolymers

## Abstract

Food packaging plays a fundamental role in the modern food industry as a main process to preserve the quality of food products from manufacture to consumption. New food packaging technologies are being developed that are formulated with natural compounds by substituting synthetic/chemical antimicrobial and antioxidant agents to fulfill consumers’ expectations for healthy food. The strategy of incorporating natural antimicrobial compounds into food packaging structures is a recent and promising technology to reach this goal. Concepts such as “biodegradable packaging”, “active packaging”, and “bioactive packaging” currently guide the research and development of food packaging. However, the use of natural compounds faces some challenges, including weak stability and sensitivity to processing and storage conditions. The nano/microencapsulation of these bioactive compounds enhances their stability and controls their release. In addition, biodegradable packaging materials are gaining great attention in the face of ever-growing environmental concerns about plastic pollution. They are a sustainable, environmentally friendly, and cost-effective alternative to conventional plastic packaging materials. Ultimately, a combined formulation of nano/microencapsulated antimicrobial and antioxidant natural molecules, incorporated into a biodegradable food packaging system, offers many benefits by preventing food spoilage, extending the shelf life of food, reducing plastic and food waste, and preserving the freshness and quality of food. The main objective of this review is to illustrate the latest advances in the principal biodegradable materials used in the development of active antimicrobial and antioxidant packaging systems, as well as the most common nano/microencapsulated active natural agents incorporated into these food-packaging materials.

## 1. Introduction

The quality and safety of food products has always been a concern in the food industry. The large number of people affected by foodborne illnesses, about 600 million people in 2015 [1], and the increasing risk of the transmission of these diseases by the growth of international trade elucidates the necessity of improving food safety strategies. Microbial spoilage causes the loss of more than 25% of food products before consumption and wastes a considerable amount of food each year [2]. Using active packaging and antimicrobial additives for food preservation are two important target areas to protect and extend the shelf-life of perishable foods by preserving them from external environmental impacts and contamination [3]. Packaging can reduce or prevent foods from physical damage and spoilage and preserve beneficial constituents and organoleptic properties from the time of packaging to the time of consumption [4]. Different parameters are considered to control the food quality, including water activity, pH, temperature, light, and the partial pressures of oxygen and carbon dioxide. Thus, food packaging can be designed based on these parameters to avoid contamination and deterioration [5]. Despite many advanced technologies in packaging, there is always more demand for novel packaging systems with a higher efficacy, which help maintain the safety and quality of food products in a safe and environmentally sustainable manner.

There are three issues in this regard. First, today’s lifestyle calls for packaged foods with an extended shelf life, especially in industrialized countries. Second, consumers are more concerned about good eating habits and the harmful effects of synthetic/chemical additives in food products. They want to consume healthier and more natural foods. Third, the rise in plastic pollution is a global concern. Therefore, the packaging should be designed following these three principles.

Among the different types of food packaging, the conventional way of packaging perishable foodstuffs, which has been extensively used for many years, is the use of plastic films based on petrochemical polymers. Considerable amounts of the polymers produced each year are used in food packaging. It accounts for 36.0, 40.7, and 46.5% of the world, Japan, and South Korea, respectively [6]. The main plastic packaging materials used in food packaging are polyethylene (PE), polyethylene terephthalate (PET), polypropylene (PP), polystyrene (PS), and polyvinyl chloride (PVC), which represent more than 90% of the total volume of plastics used in industry and about 50–70% of the total plastic waste derived from them [7,8,9].

Flexible packaging plastics are massively used in packaging due to their capacity to extend self-life and facilitate product handling. They take various forms, including plastic films, bags, flexible food packaging plastics (including mono-layered and multi-layered), and other single-use flexible plastics [6].

These materials have various advantages, such as good processing properties, excellent physicochemical characteristics, stability, resilience, and low price [10]. However, they are non-renewable, non-biodegradable, and take a long time to decompose [11]. Only 9% of these materials are recycled, 12% are incinerated, and the rest are discarded into nature [12]. Millions of tons of plastics are wasted, with more than 32 million tons yearly in only the United States [13], leading to a significant amount of plastic in the environment. Plastics accumulate in landfills and ecosystems such as oceans and coasts, causing harmful environmental effects. For example, plastic packaging can break down into microplastics with low degradation rates and find its way into fish stomachs and, therefore, into the marine food chain [14,15]. This environmental pollution poses a serious threat to animals and humans with serious consequences such as contamination of food and lack of essential nutrients [15], brain damage, and behavioral disorders [16], altering human chromosomes and obesity, infertility, and cancer after long exposure [17,18].

Furthermore, these plastic materials depend on the limited petroleum resources [19,20]. Therefore, there is an urgent need to use alternative packaging materials to overcome these drawbacks. Thanks to many recent studies, biopolymers were introduced as biodegradable packaging materials to replace the petrochemical materials. They have several advantages for manufacturing food packaging, including biodegradability and nontoxicity [21,22]. Nevertheless, there are some challenges to coping with this route, such as non-sufficient mechanical and barrier properties compared with conventional fossil-based plastics.

**Figure 1:**
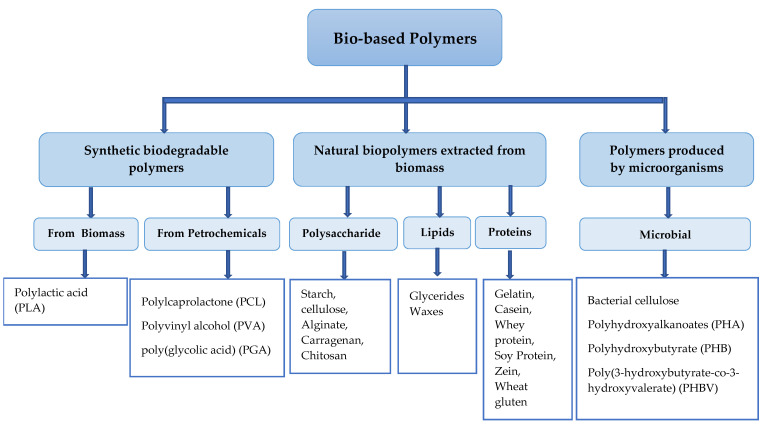
Classification of biodegradable polymers based on their source.

The degradation of biopolymers is performed by microorganisms (e.g., bacteria, fungi) throughout enzymatic catalysis processes [23]. Bio-based polymers can be classified into different categories, as shown in Figure 1. The main biopolymers used in the manufacture of food packaging are bio-based polymers derived from polysaccharides, proteins, and lipids [24]. In recent years, many studies have been conducted using these natural biopolymers extracted from biomass to produce film packaging, which results in understanding their technical characteristics and their applications as sustainable and eco-friendly materials. Their beneficial properties, such as being biodegradable, renewable, and abundant in nature, make them a good choice for use in food packaging. However, certain improvements need to be made in areas such as the mechanical properties, heat transfer, gas and water vapor permeability, and their plasticity to be competitive with plastic films [22]. Microorganisms can also produce bio-based polymers, and new technologies are able to produce synthetic biodegradable polymers, as shown in Figure 1.

The use of bio-based polymeric materials is part of the progress of food packaging that takes into account environmental concerns. The next step in improving food packaging is to prevent or retard the deterioration of packaged food. Based on the sources used, we can differentiate between first-, second-, and third-generation feedstocks based on the classification of biofuels. The first generation is based on edible materials such as maize, sugarcane, whey, or corn. The second generation includes lignocellulosic and non-edible residues such as municipal waste or side-stream products from food and agriculture industries. The third generation includes biomass from algae [25]. It has been proven that films derived from biopolymers have an excellent potential to contain various additives, such as antimicrobial and antioxidant agents, in their matrix and release them during storage [2,26,27,28]. This packaging system is called “active packaging” and helps to extend the shelf life of food by absorbing (scavenging compounds) or diffusing (emitting compounds) various compounds. Scavengers have the ability to remove undesirable substances such as oxygen, moisture, ethylene, carbon dioxide, and odor from the internal packaging space. Emitters can release substances that have a positive impact, such as antimicrobial or antioxidant agents, enzymes, nutraceuticals, and aromatic compounds [2,29].

Active packaging can reduce the risk of foodborne pathogens and improve the quality and safety of food products [1,4]. They offer many advantages by reducing, restricting, or inhibiting the growth of spoilage and pathogenic microorganisms in food products and effectively extending the shelf life of packaged content. This strategy avoids adding the antimicrobials directly to the food product and offers the potential to control their release during storage time [27,28,30].

Among the incorporated additives, much attention has been paid to natural antimicrobial agents. Among these, essential oils (EOs), which are aromatic substances that are considered as secondary metabolites and are extracted from different parts of plants and used for many years in traditional medicines, have attracted a great deal of attention in the food industry [31,32].

Despite the promising results of EOs as antimicrobial agents, their volatility, low solubility in water, and sensitivity to oxidation limit their applications in producing food packaging films [3,33]. A promising technology to eliminate these limitations is encapsulation. Encapsulation could be performed through different techniques in which EOs are considered a core material covered by wall materials. Various wall materials, such as carbohydrates, proteins, and lipids, were used to encapsulate active EOs molecules, which improves their solubility and stability and protects them during film making [31,34]. In this context, it is important to choose appropriate wall materials compatible with core properties and the conditions of encapsulation and film formation [35]. Microencapsulation is a process in which active molecules are loaded into micro-size-capsules (>1 µm) protect and isolate them from environmental conditions, masking undesirable flavors, and increasing their solubility and stability [36]. These microcapsules can be modified by reducing their size to obtain nano-size-capsules (less than 100 nm) in order to improve their properties, including bioavailability, solubility, and adsorption [34,37]. Generally, the main outcomes of nanoencapsulation are the reinforcement of biological activity and bioavailability due to the larger surface of interaction with food and, thus, the higher possibility of penetration into the cell membrane of microorganisms, which results in being more effective and supplying controlled release to the food. The strategy of incorporating nanoencapsulated bioactive molecules into film packaging extends the time of food storage by inhibiting the growth of pathogenic and spoilage microorganisms, along with using fewer amounts of antimicrobial agents and avoiding their direct addition to food products.

Considering the above discussion, this review aims to summarize the potential of bio-based polymers and the natural antimicrobial and antioxidant agents in the area of biodegradable active packaging. It also highlights the encapsulation strategies used to overcome the bioactive molecules limitations, and the most common nano/microencapsulated active natural agents incorporated into these food-packaging materials.

## 2. Biodegradable Food Packaging

More than 40% of the petroleum-based plastic materials produced are used for packaging, and half of those are used for food packaging. About 95% of plastic packaging is discarded after the first use [38]. The degradation of plastics can occur through chemical, physicochemical, or physical processes. It may take place following one or a combination of four mechanisms in nature, namely, photodegradation, hydrolysis, thermo-oxidative degradation, and biodegradation [39]. Photodegradation caused by UV-B radiation often initiates the degradation of common plastics. Degradation then moves forward through hydrolysis and thermo-oxidative degradation. Finally, degradation processes break down plastics into fragments with a lower molecular weight, which can be metabolized by microbes. The duration of degradation depends on the conditions and type of material, but the process is slow, and it may take more than 50 years for full degradation [39,40].

The definition of biodegradable bioplastic according to the European standard EN 13432 is “Under the action of microorganisms in the presence of oxygen, decomposition of an organic chemical compound into carbon dioxide, water and mineral salts, other elements present (mineralization) and appearance of a new biomass. In the absence of oxygen, decomposition into carbon dioxide, methane, mineral salts, and creation of a new biomass” [41]. Plastic biodegradation has been studied by focusing on the isolation of individual microorganisms that are able to decompose biodegradable plastics. It has been proved that bacterial and fungal consortia have a great potential in plastic waste biodegradation and bioremediation [17]. The polymeric chains are broken down into compounds such as oligomers, dimmers, and monomers with a lower molecular weight by enzymatic fractionation [42].

Plastic production has almost doubled during the last decade, and it is predicted that it will increase more, reaching 33 billion tons by 2050. The recent world health crisis caused by the spread of COVID-19 caused more plastic pollution due to the massive consumption of plastics for medical use and personal protective equipment, as well as using plastic shopping bags to prevent cross-contamination. For instance, medical waste has sharply increased by 370% and packaging plastic by 40% [43].

Consequently, plastic pollution has become one of the most critical environmental problems. Plastic has become ubiquitous, and it is a serious global problem for nature, human health, society, and the global economy [44,45,46]. Plastic pollution causes hazardous impacts on water systems, soil conditions, and air quality. The digestion and entanglement of plastic debris is an acute danger to animal and human life, and plastic waste destroys wildlife habitats [46]. Plastic consumption causes many troubles, including behavior disorders, dietary dilution, obesity, altering human chromosome sequences, infertility, and even cancer [17]. In addition, recent research investigated the effect of plastic consumption and proved the negative impacts on adolescent mental health and behavioral problems [47].

Thus, the growing accumulation of plastic wastes in nature increases the pressure to tackle the problem. Recycling aims to consume fewer raw materials, but it remains technologically and economically a big challenge [48]. Various degrees of recyclability of different products, consumer information about different recycling labels for plastic, appropriate technology, plastic waste management strategies, and risk of contamination make the recycling process complex. Indeed, the collecting, preprocessing, and recycling of plastic waste is complicated and expensive [38]. In fact, less than 10% of all plastic waste was recycled by the end of 2015 [12,49]. Incineration is also used to tackle plastic pollution, but it causes the release of undesirable gases such as dioxins, dioxin-like compounds, carbon monoxide, nitrogen oxides, alkenes, alkanes, aromatic, and chlorinated hydrocarbons into the atmosphere [50].

Therefore, biodegradability is a critical point that should be considered. Indeed, some petroleum-derived compounds, such as aliphatic-aromatic copolymers, have the potential to produce biodegradable polymers with good technical properties and modify their microstructure and composition to meet the particular requirements for different applications [51]. While biodegradability is a useful feature of these petroleum-based polymers, the processes of their production are polluting. Furthermore, fossil resources are limited, and they recharge only after millions of years. Thus, an alternative to fossil-based polymers is essential.

**Figure 2:**
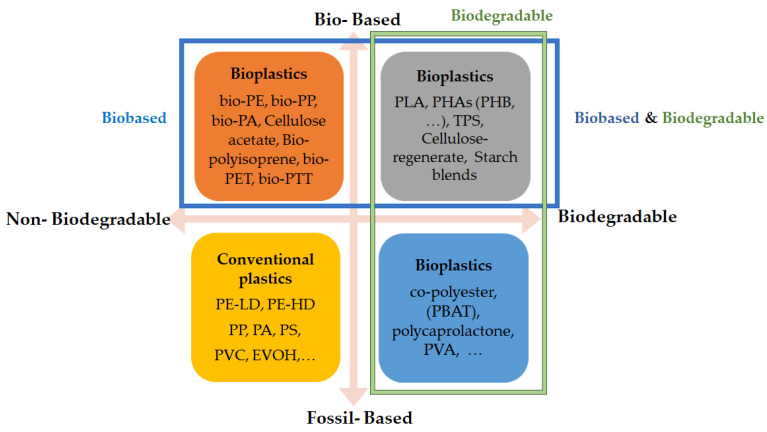
Material coordinate system of bioplastics (adapted from [53]). EVOH: ethylene-vinyl alcohol; PA: polyamide; PBAT: polybuthylene adipate terephthalate; PE: polyethylene; PE-HD: high-density polyethylene; PE-LD: low-density polyethylene; PET: poly(ethylene terephthalate); PHA: polyhydroxyalkanoate; PHB: polyhydroxybutyrate; PLA: polylactic acid; PP: polypropylene; PS: polystyrene; PTT: polytrimethylene terephthalate; PVA: polyvinyl alcohol; PVC: polyvinyl chloride; TPS: thermoplastic starch.

Thanks to extensive research, bioplastics have been introduced as a promising alternative that can be biodegradable and renewable. Figure 2 illustrates a general classification of biodegradable and non-biodegradable fossil and biobased plastics. Hence, there are the following four different groups of plastics:

- Fossil-based and non-biodegradable: refers to classical plastics such as conventional polyethylene (PE) and polystyrene (PS);
- Fossil-based and biodegradable: includes polycaprolactone (PCL), polybutylene succinate (PBS), and poly (butylene adipate-co-terephthalate) (PBAT);
- Bio-based and non-biodegradable: bio-polyethylene (PE) is an example of this group produced from bioethanol fuel, which is produced from sugar cane;
- Bio-based and biodegradable: this group is an interesting choice with high potential to apply in food packaging without environmental impacts, which can be natural or synthetic such as cellulose, starch blends, and polyesters such as PLA and PHA [52].

European Bioplastics introduced the term “bioplastics” in 2016 [52]. In order to better understand this concept and clear up any confusion, it is worth noting some definitions related to this notion. The term “biomaterials” is reserved for materials for medical use and is defined as “non-living material used and designed to interact with biological systems”. Biopolymers are polymers made by living organisms (proteins, polysaccharides, etc.) or biodegradable synthetic polymers. Agro-polymers are also applied as polymers made by living organisms and derived from agricultural resources (starches, cellulose, etc.). Materials that are derived in whole or in part from biomass resources are called “bio-based.” Biomass resources are organic materials that are available on a renewable or recurring basis, such as crop residues, wood residues, grasses, and aquatic plants. Corn ethanol is a well-known example of a bio-based material derived from biomass resources. Bio-based refers technically to any product that contains some amount of bio-based material. The term is typically applied only to materials containing carbon. Bioplastics is a broader term, dedicated to biodegradable and/or bio-based polymers produced by synthesis [52].

**Figure 3:**
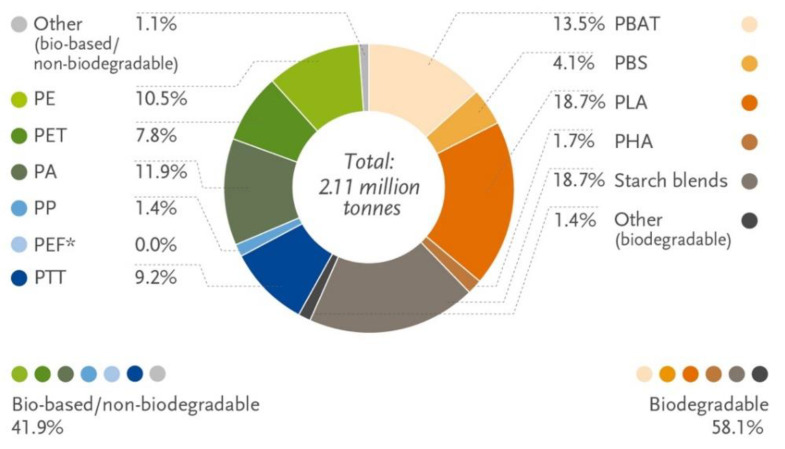
The projected global production of bioplastics in 2024 [53]. * PEF is currently in development and predicted to be available on a commercial scale in 2023.

The production of bioplastics has increased in recent years. About 2.1 million tons were produced in 2019, according to European Bioplastics, and this amount is predicted to rise to 2.4 million tons around 2024. Biodegradable and non-biodegradable parts account for 58.1% and 41.9% of total bioplastics, respectively (Figure 3). Meanwhile, the total capacity of biodegradable polymers is still quite small compared to petroleum-based plastics.

Polybutylene adipate terephthalate (PBAT), polylactic acid (PLA), polyhydroxyl alkenoate (PHA), and polybutylene succinate (PBS) are principal synthetic bioplastics commercially used to date [52]. Research on bioplastics is in progress to improve their properties, such as film-forming and barrier properties, and broaden their applications. For example, polylactic acid (PLA) is an important example of this group. It is a very versatile polyester and the most prevalent polymer synthesized from biomass. PLA has, besides starch blends, the highest production capacity of the biodegradable materials available [54]. PLA is biodegradable and industrially compostable and is used in food packaging due to its non-toxicity. It is obtained from lactic acid during the fermentation of renewable crops such as sugar beets and corn [23]. PLA is sealable at lower temperatures and acts as both a flavor and an odor barrier for foodstuffs. Its industrial composting starts after only 8 days at temperatures between 50–60 °C and 60% relative humidity (RH). This time can be decreased if PLA is blended with PCL, a biodegradable petroleum-based polymer, and becomes home-compostable [55]. All polymer materials used for packaging can be theoretically substituted with those types synthesized from renewable monomers. However, there is an economic challenge regarding processing costs and also the lack of a collection channel for these bioplastics [56].

Some of the commercially produced biodegradable polymers are shown in Table 1.

### 2.1. Biopolymers 

Biopolymers are interesting candidates for biodegradable packaging. They are extensively used on the market, especially in food packaging, because they are bio-based, nontoxic, and biodegradable. They are biodegradable materials with a faster generation rate. Biopolymers can be obtained from various resources, such as plants and domestic and marine animals. Based on their origin, they are classified into the following three main groups: polysaccharides, proteins, and lipid-based packaging [57].

#### 2.1.1. Polysaccharide-Based Packaging

Polysaccharides are one of the most important bio-based polymers. Thanks to many favorable properties and being nontoxic, polysaccharides have been used in food packaging. Polysaccharides are the most abundant carbohydrates found in nature. They are long-chain polymeric carbohydrates composed of monosaccharide units bound together by glycosidic linkages. They are made from nearly 40 different monosaccharides with various structures. There are different types of polysaccharides with distinct properties [58]. The following two classes of polysaccharides are available: (i) hetero-polysaccharides, which may contain two or more different monosaccharides such as gellan and xanthan, and (ii) homo-polysaccharides, which contain one type of mono-saccharide such as pullulan, curdlan, levan, and bacterial cellulose with linear chains [59]. Some polysaccharides, including starches, cellulose, alginates, carrageenan, and chitosan, are used for packaging because their properties are appropriate for manufacturing film packaging [4].

##### Starches

Starches are formed by large glucose units joined by glycosidic bonds with the chemical formula of (C_6_H_10_O_5_)_n_. It is the most common carbohydrate in human diets. Native starch consists of two types of glucose polymers, namely, amylose and amylopectin, and it is produced by most green plants, including corn, wheat, potatoes, rice, and cassava, etc., contributing to 80% of total starch production [60,61,62]. Starch is widely studied as a natural biodegradable material and has many applications in the fields of drug delivery, tissue engineering, biomedical scaffolds and stents, and food packaging [63]. Starch is non-toxic, abundant, biocompatible, and has good film-forming potential. Indeed, its polyhydroxy structure facilitates modulating its structure and functional properties through chemical or enzymatic reactions [64]. This great potential and the other attractive advantages of starches, such as being edible, available, low cost, and biodegradable, make them a good choice for food packaging. Despite all these properties, there are some limitations to applying native starch for food packaging, such as water susceptibility, brittle mechanical behavior, poor barrier properties, and trivial resistance to extreme processing conditions such as high temperature and shear in the native form. Several methods have been proposed to overcome these limitations. Using plasticizers such as glycerol or polyglycerol [65,66,67] and other additives, such as cellulose, gelatin, chitosan, and citric acid, can enhance the functionality of starch-based biodegradable materials [64]. Starch film properties could also be modified by using deep eutectic solvent [68] and under reactive extrusion (REx) conditions (high pressure and temperature, and low moisture content) [69]. Blending starch with many synthetic biodegradable polymers, including polylactic acid (PLA), polyvinyl alcohol (PVA), polycaprolactone (PCL), polybutyl succinic acid-butyl adipate (PBSA), and polyadipate butylene terephthalate (PBAT), is also a promising technique for improving mechanical and processing properties as well as the water-resistance of starch-based biodegradable materials [64]. These blends with starches, made by a proportion of about 30–80% of copolymers based on their application, are completely biodegradable [55,58,62,63].

Starch-based films can be used as a monolayer or laminated with other films to improve the barrier properties. Moreover, when combined with flexible polyesters such as PBAT, starch-based films become flexible, and blending with polylactic acid (PLA) improves their thermoforming properties and rigidity [57]. Furthermore, recently, the use of nanoparticles such as nanoclay or zinc in starch films has improved their mechanical properties [70,71,72].

Many authors have investigated the potential of using starch films in active packaging and reported the antimicrobial activities of different kinds of starch-based films by incorporating antimicrobial agents into them [73]. For instance, adding cinnamon essential oil to cassava starch film preserved bakery products against fungi and extended their shelf-life [70,74,75]. To date, starch-based films are emerging as an excellent alternative to conventional plastic for a variety of food packaging applications. For example, cassava starch-based foams were incorporated with grape stalks for the packaging of English cakes [76]. Starch-based films incorporated with citric pectin and feijoa peel flour (FPF) were used for apple packaging [77]. Cowpea starch-based films incorporated with maqui berry extract were used for salmon packaging [78]. Yam starch-based films incorporated with eugenol were used for pork [79], and pea starch (PS)/polylactic acid (PLA)-based bilayer films for cherry tomatoes [80]. Rye starch films containing rosehip extract (RHE) for chicken breast packaging [81], acetylated cassava thermoplastic starch and green tea blends with linear low-density polyethylene (LLDPE) films for sliced bacon [82], corn starch and gelatin films containing N-α-lauroyl-l-arginine ethyl ester monohydrochloride (LAE) for chicken breast fillets [83], and cassava starch-based films incorporated with zinc- nanoparticles for tomatoes preservation [84].

##### Cellulose

Cellulose is a polysaccharide with the formula (C_6_H_10_O_5_)_n_, consisting of a linear chain of several hundred to many thousands of β(1 → 4) linked D-glucose units. Cellulose and its derivatives are the most abundant biopolymers and are found in many resources [85]. These resources include wood, agricultural residues, factory and food wastes, food leftovers, marine organisms, some types of grass, cereal brans and husks, sugarcane bagasse, corn kernels, along with microbial biosynthesis (algae, fungi, bacteria) and peels of different fruits and vegetables [86,87]. Some of its derivatives are cellulose acetate, sulfate, nitrate and carboxymethyl, ethyl and methyl nano-cellulose [88]. They have interesting characteristics such as being edible, biodegradable, lightweight, nontoxic, bioavailable, and having nutritional value and favorable sensory and organoleptic properties such as color, appearance, aroma, flavor, and taste, and they can be easily found in significant quantities at a low cost. They also have a high potential to be used for encapsulating and incorporating various active molecules of antimicrobial and antioxidant materials [89]. It is worth mentioning that cellulose of bacterial origin exhibits extraordinary and differentiated properties compared to other polysaccharide-based polymers. It is receiving extensive attention for applications in the food industry as a thickening and gelling agent, stabilizer, water-binding additive, and also as a food packaging material [90].

Cellulose has excellent physical and mechanical properties and high thermal resistance, but there are some shortcomings, including a high water absorption capacity and insufficient interfacial adhesion. Cellulose has been studied for many years to improve its properties. Cellulose multilayers were assessed to enhance barrier properties, resulting in further tensile strength, as well as grease resistance and barrier to water [91]. The effect of incorporation of carotenoids in cellulose acetate, as natural antioxidants, was studied and showed good protection for foods susceptible to photooxidation [92]. Thereby, due to its outstanding characteristics, cellulose used for food packaging reduces the cost of food packaging and preserves a wide range of foodstuffs in an eco-friendly manner.

##### Alginate

Alginate is the salt of alginic acid, a bio-based polymer consisting of D-mannuronic and L-guluronic monomers extracted from brown marine algae (brown seaweeds) and some bacteria, such as *Pseudomonas aeruginosa* [93,94]. Alginate is nontoxic, non-antigenic, biocompatible, and biodegradable, and it has the capacity to form a hydrogel when applied in encapsulation. Due to these properties, alginate is used in textiles, cosmetics, and pharmaceutics, and also in food industries as a thickener and stabilizer [95,96,97,98]. In the food industry, alginate is extensively used in the preparation of edible coatings because of its film-forming properties, hydrophilicity, and biocompatibility [99,100]. The application of alginate-based coatings in food preservation is limited due to their poor antimicrobial, UV-shielding, and water barrier properties. Some studies were dedicated to overcoming these shortcomings [101,102]. One recent study investigated the effect of the incorporation of phenolic compounds such as thymol into the sodium alginate film for fresh-cut apple slices. This film exhibited remarkable inhibition of the growth of *Staphylococcus aureus* and *Escherichia coli*, as well as a reduction in weight loss and retention of nutrients, and the surface color of apple slices. These thymol/sodium alginate films also showed a high tensile strength and elongation at break, as well as UV–vis light-blocking capability [103].

##### Carrageenan

Carrageenan is a polysaccharide extracted from marine red algae by hot alkali separation. Akin to other polysaccharides, carrageenan is nontoxic, biocompatible, and biodegradable with a low immunogenicity. Furthermore, carrageenan offers excellent properties compared with other encapsulation materials, such as a better stability of capsules, higher electronegativity, and a better protection of encapsulated contents, allowing them to be considered a good candidate in delivery systems for bioactive ingredients [104,105]. Therefore, it has an extensive range of applications in the food and pharmaceutical fields. Various structures of carrageenan make different biological activities, including antioxidant, antitumor, immunomodulatory, anti-inflammatory, anticoagulant, antiviral, antibacterial, antifungal, and anti-hyperglycemic properties.

Carrageenan has many applications in the food industry, including gelling, thickening, stabilizing, protective coating, and fat substitution [106]. Many authors developed pH-sensitive and antioxidant-packaging films based on carrageenan [107]. Another investigation showed a protective effect of fish oil, encapsulated in carrageenan, against lipid and protein oxidation and an improvement in the shelf life and sensory characteristics of enriched nuggets [108].

##### Chitosan

Chitosan, a linear polysaccharide with (1-4)-linked 2-amino-deoxy-β-d-glucan derived from chitin, is the second most abundant biopolymer and the most abundant biopolymer of animal origin. It is found in the exoskeletons of crustaceans and insects and in the cell walls of fungi and yeast [109]. It is a multipurpose biopolymer with interesting physicochemical and biological properties that are applied in agriculture, pharmacy, and biomedicine industries [110]. It is used in food packaging due to its low cost, biodegradability, biocompatibility, non-toxicity, film formation, viscosity, and ion binding [111]. The combination of chitosan with other biomaterials, nanometals, and active compounds allows further exploitation of chitosan and modification of some of the shortages of its characteristics, including hydrophilicity, weak mechanical properties, low gas permeability, and low encapsulation efficiency, as well as enhancing the bioavailability and biological properties [112].

The emerging chitosan-gelatin composite films present excellent physical properties, including mechanical, surface hydrophobicity, color, barrier, and thermal characteristics [113]. Further, chitosan films incorporated with ε-polylysine were used for packaging beef fillet. Different features were investigated, and this technique was introduced as an effective way to extend the shelf life of beef fillets and maintain their quality during refrigerated storage [114]. Another study highlighted the antioxidant and antimicrobial activities of chitosan-based packaging incorporated with nanocapsules of *Cinnamodendron dinisii* essential oil with zein as the wall material. This active chitosan-based film was tested on beef and showed preservative effects against spoilage and color changes [115].

#### 2.1.2. Protein-Based Packaging

One of the other important types of bio-based polymers are proteins with two main plant and animal origins. They are made of different polar and nonpolar α-amino acids. There are different sources of plant-origin proteins such as wheat gluten, corn zein, soy, peanut, rice-bran, cotton seed, barley, and sunflower, along with animal origins such as gelatin, collagen, casein, whey, and fish myofibrillar protein [116]. The proteins of plant origin are used more due to their greater availability and lower cost.

Proteins are extensively used as packaging materials due to their good mechanical characteristics, abundance, and high nutritional value. They also have the potential to be incorporating agents for active packaging applications in addition to their nontoxicity and biodegradability [116,117]. However, some shortcomings, such as a high level of sensitivity to moisture and poor water vapor barrier properties, need to be improved. In fact, films that are composed of plant protein isolates have excellent oxygen barrier properties due to their polar nature and crosslinked polymer network; however, the water vapor barrier is low [25]. Using plasticizers, cross-linkers, and other additives through various physical, chemical, and enzymatic methods can be applied for this purpose [4,118]. The most used protein-based polymers are presented below.

##### Soy Protein

Soy proteins are composed of globulin proteins 7S (β-conglycinin) and 11S (glycinin), which have different structures, functional, and molecular properties. The functional properties of soy products depend on these two components [119].

Soy proteins have many applications, such as adhesives, composites, plastics, and so on, in different industries, including the food industry. Soy proteins are biodegradable and have exceptional film-forming properties. Furthermore, the effectiveness of incorporating antimicrobial compounds into soy protein films has been reported; therefore, soy protein can be used for producing film packaging [120,121,122]. Nevertheless, there are some limitations, such as low mechanical and thermal properties, poor processability, and water sensitivity, which can effectively be modified through laminating, coating with other polymers, plasticizing, nanoparticle reinforcing, or blending methods [123]. As an example, Ref. [124] investigated the mechanical and barrier properties of soy protein isolate-based films coated with polylactic acid and demonstrated improvements in the mechanical and water barrier properties of the film. Furthermore, using cellulose nanocrystals in soy protein films showed better film-forming, tensile strength, barrier properties, and water resistance. This film was evaluated for packaging pork and strawberries and showed fewer total viable counts and total volatile basic nitrogen of stored pork meat and extended the shelf-life of strawberries [125]. A new strategy of incorporating polyethyleneimine as a plasticizer and lignin-silver nanoparticles as a green reinforcer into soy protein films has made it possible to manufacture tough, strong, and UV-shielding soy protein-based composite films [126].

##### Wheat Gluten

Wheat gluten is a by-product of the wheat starch industry. It comprises mainly two water-insoluble proteins called glutenin and gliadin. Gliadins are composed of low-molecular-weight proteins ranging from 30,000 to 80,000 Da and glutenin containing high-molecular-weight proteins ranging from 80,000 to several million Da. The functional and structural characteristics of these two proteins determine the functional properties of wheat gluten. Generally, the films produced from glutenin showed better barrier properties than the films from gliadins or whole gluten [91,127,128]. Owing to its viscoelastic properties, lower solubility, and biodegradability, wheat gluten has many food and nonfood applications. Wheat gluten films are used in food packaging because of their oxygen barrier properties. However, they present a poor water vapor barrier that can be modified by adding plasticizers, coatings, and blends with hydrophobic polymers [129]. In this regard, Ref. [130] reported that wheat gluten/silica hybrid coating films could improve the inherent moisture sensitivity of this protein with a four-fold reduction in water vapor transmission rate. In addition, the films prepared from the blending of three thermoplastic gluten and polycaprolactone (PCL), with and without chrome octanoate as a food-grade catalyst, were recommended as potential shape-memory food packaging materials [131].

Wheat gluten films also have the potential for combination with antimicrobial materials to protect contents from spoilage [132]. The use of carvacrol as an antimicrobial agent and montmorillonite as a filler in wheat gluten-coated papers improved the antimicrobial efficiency against *Escherichia coli* [133].

##### Corn Zein

Zein is a class of prolamin protein found in the endosperm of corn (maize) mainly composed of α-zein, β-zein, and γ-zein [134]. It is extracted from corn gluten meal, a byproduct of starch production, through ethanolic extraction. Zein can form tough, glossy, hydrophobic, greaseproof coatings, and it is considered an antimicrobial agent. Zein is an abundant, renewable material with a low cost. Thus, it has a variety of applications in the food, chemical, environmental, medical, metallurgical, pharmaceutical, and biotechnology industries [135,136].

Zein is a hydrophobic and thermoplastic material, soluble in alcohol but insoluble in water that has film-forming properties and has been used commercially as an edible film for nuts and confectionery products for many years [4,137]. Zein films are manufactured via different processes, including the casting of solutions of zein, thermoplastic processing, and blown extrusion. The mechanical and thermal characteristics of zein are affected by the processes by which they are produced [137,138]. They offer high tensile strength and low water vapor permeability, and the water resistance of zein films can be improved by lamination with fatty acids because of their ability to form hydrophobic interactions with fatty acids [139]. Some mechanical properties of zein films need to be improved, such as fragility and flexibility, by adding plasticizers or blending with other biopolymers to remove these drawbacks [140,141,142]. A recent study assessed the mechanical properties of gelatin/zein composite films and introduced them as good candidates for high moisture food packaging applications due to the great improvement in water insolubility, water vapor permeability, and mechanical properties compared with conventional gelatin and zein films [143].

##### Casein and Whey Proteins

Milk proteins are composed of the following two dominant proteins: casein in the form of micelles, which accounts for approximately 80% of the total proteins, and the remaining 20% are whey proteins. Casein, the main protein of milk, has four types, namely, αs1-, αs2-, β-, and κ-casein [144]. Casein micelles are composed of large numbers of casein molecules, which are stabilized by calcium-phosphate bridging [145]. It can be used in food packaging because it can produce a flexible and transparent film with good gas and lipid barrier properties at low relative humidity. It also shows good emulsifying behaviors along with the mechanical and thermal stability of the caseinate.

Whey protein is a by-product of the cheese-making process or a by-product of acid casein production. Roughly 70% consists of a mixture of β-lactoglobulin (β-Lg) and α- lactalbumin (α-La). The other components of whey protein are protease peptones, bovine serum albumin, immunoglobulins, and other minor proteins [146].

Globally, milk proteins have high nutritional value, which provides much of the protein and amino acid dietary needs for the human diet. They are natural animal products with excellent functional and sensory properties and are safe for food applications [147]. Their functional and structural characteristics make them versatile materials as a sustainable source of biopolymers. Their most important properties are their nutritional value, biodegradability, biocompatibility, safety, thermal stability, emulsification, gelation, foaming, and water-binding capacities [148]. Thanks to these features, they are used in packaging and also in active packaging with incorporated bioactive and nutraceutical compounds [13,149]. The film-forming capacity and biodegradability of milk proteins make them a promising alternative to conventional non-biodegradable packaging [150]. Composite films containing milk protein-based biopolymers degrade in a very short time, with 100% degradation on day one without harmful residues. However, there are economic feasibility challenges to be overcome in the coming years [151].

Despite the potential of using milk proteins as a biopolymer for packaging, they exhibit some disadvantages compared to other conventional polymer materials, including their poor mechanical and barrier properties against gases, aromas, and water vapor. These drawbacks can be overcome in several ways, including blending with other edible biopolymers to form composite materials [152], multilayering films by coatings with nano- or micro-dimensions of more than one type of biopolymer, and also adding synthetic plasticizers [144,153]. An improvement in the bactericidal properties of the casein and chitosan blend in film packaging due to the presence of ionic interaction between both macromolecules was proven by [154].

It is worth noting that milk proteins constitute a range of biological activity components. For example, casein is an important source of antimicrobial peptides [155,156]. Furthermore, milk proteins can be used as macro and nano carriers for the encapsulation of nutraceuticals, vitamins, minerals, and antimicrobial compounds due to their biocompatibility, ease of controlling the release, and dispersibility of the encapsulated compounds [157]. Using antimicrobial agents in casein and whey protein film packaging has been investigated in many studies. The inhibitory effect on *Staphylococcus aureus* of casein-based films containing *Zataria multiflora* as the essential oil has been proved [158]. Further, the antimicrobial potential of cheese whey bioactive proteins and peptides such as lactoferrin in the development of antimicrobial edible film composites was reported by [159].

##### Gelatin

Gelatin is produced from the partial hydrolysis of collagen. In the past, gelatin has been manufactured from bovine and porcine skin and bone. In recent years, fish and poultry have also been used to produce gelatin. Production of fish gelatin is increasing due to vegetarianism and the potential of halal and kosher markets [160]. Gelatin has gel-forming properties that offer many applications in food, photography, cosmetics, and pharmaceutics. More notably, it is widely used in food to provide elasticity, viscosity, and stability. Further, it has a linear structure and limited monomer composition, leading to excellent film-forming properties [161]. Gelatin is used as an emulsifier, foaming agent, colloid stabilizer, biodegradable film-forming material, micro-encapsulating agent, and source of bioactive peptides when enzymatically hydrolyzed [162].

Gelatin films possess good mechanical, functional, and barrier properties, depending on the film’s formulations and processing conditions. However, they are sensitive to moisture and show poor barrier properties against water vapor, which can be improved by using plasticizers or cross-linking agents or combining with other biopolymers such as soy protein isolate, oils, fatty acids, and certain polysaccharides [163,164,165,166].

Adding antimicrobial and antioxidant compounds into gelatin films also conferred more preservation of foodstuffs. Recently, the effect of curcuma in gelatin film was investigated by [167] and revealed remarkable antimicrobial activity against foodborne pathogenic bacteria, *E. coli*, and *L. monocytogenes*, and showed strong antioxidant activity along with improvement of the UV protection, water vapor barrier, and mechanical properties. In addition, the antimicrobial and antioxidant properties of the gelatin films were increased by embedding different kinds of functional nanoparticles such as quercetin, lactoferrin, and chitosan nanofiber into a gelatin-based film [168].

In conclusion, all these protein-based biopolymers are being studied massively due to their promising functions for the production of biodegradable active packaging with many beneficial economic and environmental attributes, enhancing the safety and quality of food products.

#### 2.1.3. Lipids-Based Packaging

Lipids are classified into different types, such as phospholipids, phosphatides, mono-, di-, and tri-glycerides, terpenes, cerebrosides, fatty alcohol, and fatty acids. They are extracted from various natural resources of animal, insect, and plant origins. Among them, glycerides or waxes are mainly used in the production of films. These compounds are mainly nonpolar with a high hydrophobicity, insoluble in aqueous media, and soluble in organic solvents. They are used for coatings and edible films based on different biodegradable materials, including polysaccharides and proteins, due to their excellent moisture barrier properties [13,169]. They have a glossy appearance, minimize moisture loss, and reduce the cost of packaging films [170]. Lipids are also used as carriers for various bioactive compound-delivery in the food and pharmaceutical industries [171,172].

Packaging films using lipids have been studied for many years, and it is moving forward because of the capacity of lipids-based polymer to produce effective active edible films. Palm wax was incorporated into fish gelatin film used in food packaging, resulting in better UV barrier, mechanical, and water resistance properties [173]. Lipids can also be combined with other biopolymers in order to modify their poor hydrophobic characteristics. Recently, carnauba wax was added into a starch-based film to enhance its hydrophobicity and reduce its water solubility and improve its thermal stability and moisture, light and water vapor barrier properties [174].

#### 2.1.4. Microorganism-Based Packaging

Microorganisms are another source to produce biopolymers. Many microorganisms are used to produce polymer materials, including *Alcaligenes, Bacillus*, *Azotobacter*, *Rhizobium*, and *Halobacterium* [57,175]. For instance, some polymers are produced from microorganisms such as polyhydroxyalkanoates (PHA), poly(β-hydroxybutyrate) (PHB), poly(3-hydroxybutyrate-co-3-hydroxyvalerate) (PHBV), levan, curdlan, gellan, dextran, bacterial cellulose, and microbial polysaccharides including exopolysaccharides, capsular polysaccharides, and xanthan. They are applied in the medical, agricultural, and packaging fields. Among them, PHAs and bacterial cellulose are the most produced polymers [22,55]. PHA polymers are resistant to heat and ultraviolet light. PHAs are durable for high-speed processing and can withstand high temperatures during storage [25]. Genetically modified microorganisms were also introduced to produce biodegradable polymers with renewable sources. However, genetic engineering is controversial, and it is believed that it is harmful to human health.

## 3. Active Biodegradable Packaging Films

Packaging is a barrier from gases, moisture, dust, or light, but conventional packaging is not totally effective for preventing the spoilage of food products [28] by chemical or biological reactions. Chemical spoilage mostly happens by oxidation processes of food ingredients, and biological spoilage is caused by enzymes, viral and parasitic activity, and microbial contamination [176]. Among them, microbial contamination is one of the main reasons for food deterioration. Many preservation techniques have been applied for a long time, such as fermentation, drying, thermal processing, freezing, refrigeration, modified atmosphere, irradiation, and, more notably, adding antimicrobial agents to the food [177].

In this regard, antimicrobial agents can be mixed with food to inhibit microbial growth. In such cases, a considerable quantity of antimicrobial compounds would be required, which may have an undesirable impact on the quality, taste, organoleptic properties, and appearance of foods. Moreover, as many of these materials have low stability, there is the possibility of neutralization of antimicrobial agents quickly at the same time by the active substances in food or during processing. Antimicrobial agents can also be added directly to the surface of food, and their effectiveness can be limited by diffusing them into the mass of food, which can cause the same unfavorable results as their combination with food [176].

Therefore, in order to overcome these problems and extend the preservation period of foods along with maintaining their quality, their sensory property, and freshness, some modern strategies are needed. Intelligent packaging and active packaging are two promising technologies that promote the shelf life of food products with minimal adverse effects. In intelligent packaging, various sensors and indicators are employed, including time-temperature indicators, gas indicators, humidity sensors, optical, calorimetric, and electrochemical biosensors for detecting defects, quality monitoring, and following the packaged contents to control the storage conditions during all steps of food processing to the consumption step [29].

Active packaging applies absorption and diffusion mechanisms to protect the foodstuffs and extend their shelf life. Active packaging can act as a scavenger and absorb various materials such as carbon dioxide, oxygen, and ethanol, which cause the spoilage of foods. Active packaging can contain various active molecules such as antimicrobials, antioxidants, and even coloring and nutritional agents, which can be emitted from the packaging into the foods during the storage time [178].

Active and intelligent packaging are already used for food packaging in the USA, Japan, Australia, and other countries. California-based Nature Fresh Farms is a company that uses active packaging for vegetables such as brussels sprouts and cucumbers, with plans to widen active packaging use for their products. Some other companies, including Uvesa, ElPozo Alimentación, BTSA. Biotecnologías Aplicadas, Monteloeder, Nurel, Bandesur Alcalá, and SP group, collaborated in an active packaging project to extend the shelf-life of meat with active packaging (Avanza-S project). Moreover, an edible coating developed by Eden Agritech, a startup company in Thailand, extended the shelf-life of fresh-cut fruit by up to three times [179].

However, strict European regulations for food contact materials and the safety aspects of the application of these innovative technologies have restricted their applications. Knowledge about consumer acceptance, economic aspects, and environmental impacts is required for more developments and applications [180]. Indeed, the development and application of active and intelligent packaging systems needs both legislative regulation and consumer acceptance. In this regard, a European study was conducted within the EU FAIR R&D program framework, called the Actipak project (January 1999–December 2001). It contributed to establishing and implementing active and intelligent packaging in the current relevant regulations for packaged food in Europe and initiated amendments to European legislation for food-contact materials. The aim of this project was the classification of active packaging, including antimicrobial release films, and the evaluation of antimicrobial films on cheese, meat, and fruit [180]. At that time, there was no European regulation that specifically covered the use of active and intelligent packaging. Later, in 2009, the issuing of European Regulation (EC) No. 450/2009 helped faster market penetration in Europe [181].

### 3.1. Antimicrobial Active Packaging 

Antimicrobial packaging can be made from different polymers, including petrochemical-based polymers and bio-based materials. Meanwhile, a wide range of antimicrobial agents, chemical or natural, can also be applied to manufacture antimicrobial packaging films. The necessity of producing healthy, high-quality foods and minimizing the side effects of chemical agents, along with less environmental impacts, causes researchers to use natural antimicrobial agents and biodegradable materials to make antimicrobial biodegradable packaging films [176].

Antimicrobial agents incorporated into biopolymeric compounds are released slowly into the food surface over time. In this way, an active concentration of antimicrobial agents is present on food for a longer time without the risk of neutralization and negative effects on food. This method extends the lag phases for the growth of microorganisms and prevents food spoilage. Keeping the balance between the release rate of antimicrobial agents and the food spoilage kinetics is a key point in optimizing the effectiveness of active packaging [179].

It is not possible to find a single antimicrobial agent that can suppress all types of microorganisms and prevent all kinds of food spoilage. However, many different biopolymers have potential applications in active packaging.

Table 2 summarizes the recent studies related to food preservation by using antimicrobial bio-based films. There are several antimicrobial compounds authorized based on the standards of each country.

#### 3.1.1. Natural Antimicrobial Agents of Plant Origin

Antimicrobial agents can be found in nature, especially in plants. A wide range of antimicrobial compounds are synthetized naturally in plants to protect against microorganisms and other predators.

There is a preference to use these agents in food because of the easy regulation process to extract them and consumer demand to reduce the use of synthetic food additives due to their undesirable effects. The most important natural antimicrobial agents are extracted from spices and herbs such as rosemary, cloves, horseradish, mustard, cinnamon, sage, oregano, basil, marjoram, savory, thyme, and many others. They are mainly in the form of essential oils (EOs) [182].

**Table 2:** Antimicrobial bio-based packaging for food applications.

Food	Antimicrobial Agents	Bio-Based Polymer	Target Microorganisms	Main Findings	References
**Cheese**	Essential oils from the following two spices: *Rosmarinus officinalis* and *Laurus nobilis*	Zein nanofibers	*Staphylococcusaureus* and *Listeria monocytogenes*	Both showed antimicrobial activity, with higher effects from *Laurus nobilis* than *Rosmarinus officinalis.*	[183]
**Cheese**	Moringa oil	Chitosan	*Listeria monocytogenes* and *Staphylococcus aureus*	High antibacterial activity against *Listeria monocytogenes* and *Staphylococcus aureus* at 4 °C and 25 °C for 10 days, without any effect on the sensory quality of cheese.	[184]
**Soft (minas frescal) cheese**	Nisin	Starch/halloysite/nanocomposite films	*Listeria monocytogenes*	After 4 days, antimicrobial nanocomposite films with 2 g/100 g nisin significantly reduced the initial counts of the bacterium and those with 6 g/100 g nisin completely inhibited *L. monocytogenes*.	[185]
**Cheddar cheese**	Nisin-silica liposomes	Chitosan	*Listeria monocytogenes*	Anti-Listeria activity without effect on the sensory properties of cheese.	[186]
**Fresh cheese and apple juice**	Nisin	pullulan nanofibers	*Leuconostoc mesenteroides* *L. monocytogenes* *Salmonella Typhimurium*	Bactericidal effect against *L. monocytogenes*, *L. mesenteroides*, and *S. typhimurium* in apple juice after 20, 48, and 48 h, respectively.	[187]
**Chicken meat**	Tea tree oil (TTO)liposome	Chitosan	*Salmonella enteritidis and* *Salmonella typhimurium*	Almost no impact on the sensory properties. In total, 5 log_10_ reductions of *Salmonella* were observed in chicken meat by TTO liposomes/chitosan nanofibers treatment for 4 days at 12 °C and 25 °C.	[188]
**Fish**	Bacteriocin 7293 (Bac7293), a novel bacteriocin from *Weissella hellenica* BCC 7293	Poly (lactic acid)/sawdust particle biocomposite film	Gram-positive: *Listeria monocytogenes*, *Staphylococcus aureus* Gram-negative: *Pseudomonas aeruginosa*, *Aeromonas hydrophila*, *Escherichia coli*, *Salmonella Typhimurium*	Growth inhibition on both Gram-positive and Gram-negative bacteria.	[189]
**Fish**	Essential oil from *Plectranthus amboinicus*	Chitosan	*Bacillus subtilis*, *Escherichia coli*, *Staphylococcus aureus*, *Salmonella typhimurium*, *Klebsiella pneumoniae*, *Pseudomonas aeruginosa*	Improvement in tensile strength, opacity, and water vapor barrier with antimicrobial efficiency against foodborne pathogens.	[190]
**Fish and chicken**	*Amaranthus* leaf extract (ALE)	Polyvinyl alcohol (PVA) and gelatin	Gram-positive: *Bacillus cereus* and *Staphylococcus aureus* Gram-negative: *Escherichia coli* and *Pseudomonas fluorescence*	Better protection against UV light and reduced water solubility and water vapor permeability, and improvement of mechanical properties. Inhibition of microbial growth and minimization of oxidative rancidity in 12 days shelf life compared with 3 days shelf life for neat film.	[191]
**Fish fillets**	Curcumin and nisin	Electrospun nisin/curcumin (NCL) nanomats	Lactic acid bacteria (LAB) and Total Mesophilic Aerobic (TMAB)	On the 4th day, the count of TMAB in the samples coated with NCL mats was 3.28 log CFU g^−1^ compared to 6.61 log CFU g^−1^ in control samples.	[192]
**Chicken breast fillets**	Virgin olive oilgrape seed oiland savory essential oil	Gelatin-pectin	*Staphylococcus aureus*, *Salmonella typhimurium* *Fluorescence pseudomonas*	Savory essential oil presented more antimicrobial activity. The mixture of them in film showed antimicrobial activity against mentioned bacteria for 12 days storage.	[193]
**Chicken breast fillets**	Carvacrol (0.75% *w*/*w*) and citral (1.0% *w*/*w*)	Sago starch and guar gum	*Bacillus cereus* *Escherichia coli*	The tensile strength of films reduced while elongation at break increased, and the film showed good antimicrobial activity.	[194]
	*Laurus nobilis* essential oil and *Rosmarinus officinalis* essential oil	Polyvinyl alcohol (PVOH)	*Listeria monocytogenes*	Inhibition of the lipid oxidation together with antimicrobial activity.	[195]
**Lamb meat**	2% rosemary oil	Cellulose nanofiber/whey protein matrix containing titanium dioxide particles (1% TiO_2_)	*Escherichia coli* *Salmonella enteritidis* *Listeria. monocytogenes* *Staphylococcus aureus*	The active packaging significantly reduced microbial growth, lipid oxidation, and lipolysis of the lamb meat during storage.	[196]
**Strawberries**	Cinnamon	Polybutylene adipate terephthalate (PBAT) films loaded ith cellulose nanofibers (CNF)	*Salmonella enterica* subsp. *enterica serovar Choleraesuis* and *Listeria monocytogenes*	The active film showed a high thermal stability with decreasing water vapor permeability. Strawberries had lower weight loss after 15 days of storage, better freshness preservation without fungal attack, and antimicrobial activity against bacteria.	[197]
**Cherry tomatoes**	Cinnamon	Chitosan as the outer layer and the mixture of sodium alginate and the amphiphilic starch as the intermediate layer	*Escherichia coli* *Staphylococcus aureus*,	This active film showed more freshness and lower weight loss rate within two weeks compared to polyethylene films. The inhibition growth rates for *E. coli* and *S. aureus* were 36% and 30%, respectively, and soil biodegradability rate was 70% in 28 days.	[198]
**Cucumber**	Clove oil	Chitosan	*Escherichia coli*	Maintained the color and flavor of cucumber for more than 4 days and until 4.97 log_10,_ reductions of *E. coli* biofilm in population.	[199]
**Strawberries**	*Thyme*	Porous polylactic acid (PLA) nanofibers and coated with poly(vinyl alcohol)/poly(ethylene glycol) (PVA/PEG) blends	*Escherichia Coli* *Staphylococcus aureus*	Strawberries packed with this film exhibited better freshness and more than 99% antimicrobial activity against mentioned bacteria.	[200]
**Strawberries**	*Citral Litsea* (L.) *cubeba* essential oil	Polyvinyl acetate (PVA)	*Escherichia coli* *Staphylococcus aureus* *Aspergillus niger*	The broad-spectrum, direct, and indirect (gas phase) antimicrobial activity was observed against bacteria and fungi.	[201]
**Vegetable products**	Cinnamon and oregano	Cellulose	*Listeria grayi* *Listeria monocytogenes*	Cinnamon and oregano essential oils inhibited the growth of both bacteria in the vapor phase. The packaging with cellulose stickers impregnated with cinnamon reduced the *Listeria* count on frozen vegetable samples.	[202]
**Fruit**	Cinnamon	Zein	*Escherichia coli*	Improvement of barriers and mechanical properties of zein film with antimicrobial effect on *E. coli* and fruit samples.	[203]
**-**	*Zataria multiflora* and *Cinnamon* *zeylanicum* essential oils	Soy Protein Isolate (SPI)/Gelatin	*Staphylococcus aureus* *Bacillus cereus* *Listeria monocytogenes.* *Salmonella typhimurium* *Escherichia coli*	This active film incorporated with 20% *Z. multiflora* reduced 100% of *S. aureus*, *B. cereus*, and *L. monocytogenes.* The reduction for *E. coli* and *S. typhimurium* were 70% and 63%, respectively.	[204]
**-**	Lavender essential oil	Starch, furcellaran, and gelatin	*Escherichia coli* *Staphylococcus aureus*	Increase film thickness and decrease water absorption and degree of swelling of the film with increasing concentration of oils. Additionally, the film showed both antioxidant and antimicrobial activity.	[205]
**-**	Rosemary mint essential oil, nisin and lactic acid	Chitosan, pectin, and starch	*Bacillus subtilis*, *Escherichia coli*, *Listeria monocytogenes*	Rosemary and nisin improved water barrier properties, tensile strength, and thermal stability, as well as microstructural heterogeneity and opacity. The film also showed inhibitory activity against all mentioned bacteria and antioxidant activity.	[206]
**-**	Rosemary essential oil	Chitosan	*Listeria monocytogenes*, *Pseudomonas putida Streptococcus agalactiae*, *Escherichia coli*, and *Lactococcus lactis*	Antimicrobial activity with a better effect on Gram-positive bacteria (i.e., *L. monocytogenes*, *S. agalactiae*)	[207]
**-**	Rosemary essential oil	Glycerol, gelatin, chitosan, and pectin	*Bacillus subtilis*, *Staphylococcus aureus*, *Enterococcus aerogenes*, *Enterococcus faecalis* and *Escherichia coli*	Optimization of the mixture with 10.0% of chitosan, 24.3% of gelatin, 0.5% of pectin, and 65.2% of glycerol. Inhibition of the growth of the mentioned microorganisms.	[208]
**-**	*Glycyrrhiza glabra* L. root essential oil (GGEO)	Carboxymethyl cellulose–polyvinyl alcohol (CMC-PVA)	*Gram-positive:* *Listeria monocytogenes*, *Staphylococcus aureus* *Gram-negative:* *Escherichia coli* *Salmonella Typhimurium*	Better inhibitory effects against the Gram-positive bacteria compared with Gram-negative bacteria.	[209]
**-**	Carvacrol (0.75% *w*/*w*) and citral (1.0% *w*/*w*)	Sago starch (SS) and guar gum	*Bacillus cereus* *Escherichia coli*	The tensile strength of films reduced while elongation at break increased, and the film showed good antimicrobial activity.	[194]

Essential oils (EOs) are the second metabolite and naturally volatile organic compounds produced from the extraction of different parts of plants, including flowers (jasmine, rose, violet, and lavender), herbs, buds (clove), leaves (thyme, eucalyptus, salvia), fruits (anis, star anise), twigs, bark (cinnamon), zest (citrus), seeds (cardamom), wood (sandal), rhizomes, and roots (ginger) [210]. They are present in cavities, secretory cells, epidermic cells, canals, or glandular trichomes. Essential oils present antibacterial, antifungal, anti-inflammatory, anesthetic, insecticidal, and antiviral properties. Thereby, they have many applications in the cosmetic, perfumery, pharmaceutic, beverage, feed, and food industries [211,212]. They are produced by various methods, such as solvent extraction, supercritical fluid extraction, hydro distillation, solvent-free (microwave) extraction, physical expression (cold pressing), ultrasound-assisted extraction, and combinations of extraction methods [182,210,213]. Essential oils are composed of a mixture of about 20–80 individual components at different concentrations, most of which are terpenes, terpenoids, aldehydes, isoflavonoid acids, and ketones. The antimicrobial activity of EOs depends on the chemical structure of these individual components. There are about 3000 essential oils; approximately 300 are used for food, cosmetics, fragrance, and medical applications. EOs are naturally hydrophobic and lipophilic compounds, with a density that is often lower than water. They are soluble in organic solvents such as ether, alcohol, and fixed oils but immiscible with water. They can be isolated from the aqueous phase via decantation [33,210].

Essential oils have gained a growing interest in the food industry and packaging due to their antimicrobial and antioxidant properties applied to the preservation of foodstuffs, including fruits, vegetables, fish, meat, and dairy products [3]. As they are natural compounds, they can be an excellent alternative to synthetic food additives. Their hydrophobicity allows them to permeate through the lipids of the cell membrane of bacteria, disrupting the structure and finally inhibiting them [33].

The antimicrobial effect of EOs has been studied in detail, and it depends on their chemical structure. It is confirmed that the presence of hydroxyl groups in phenolic compounds such as carvacrol and thymol has a key role in their activities against bacteria [214]. However, EOs are unstable, volatile compounds, which prevents their direct use on foods, as that would be ineffective with uncontrolled release. In fact, they start to degrade easily through oxidation, volatilization, heating, and light if they are not preserved from external factors [33,215]. Furthermore, EOs are aromatics that can have undesirable sensory effects. Encapsulation of essential oils into carriers such as liposomes, polymeric particles, and solid lipid nanoparticles is indispensable [3,216].

Many studies have been conducted on the antimicrobial activities of EOs on different microorganisms with promising results in inhibiting the growth of different types of pathogens, which has attracted huge attention in food packaging [217]. In this regard, a recent study conducted by [202] evaluated the antimicrobial activities of cinnamon, oregano, and carvacrol against *L. grayi* and *L. monocytogenes* in vitro. The results showed their effectiveness in inhibiting the growth of *L. grayi* as well as *L. monocytogenes* in the vapor phase. Chitosan films prepared by incorporation of apricot kernel essential oil, which is a strong antioxidant and antimicrobial agent, in different concentrations, showed excellent antimicrobial and antioxidant properties as compared to neat chitosan films. They successfully inhibited fungal growth on packaged bread slices, improved water resistance, and enhanced the water vapor barrier property [218].

#### 3.1.2. Natural Antimicrobial Agents of Animal Origin

Some animal proteins and enzymes can act on both Gram-positive and Gram-negative bacteria by destroying their cell membranes [219]. Antimicrobial peptides are a large group of antimicrobial agents of animal origin, including pleurocidin, lactoferrin, defensins, and protamine [220]. Some enzymes such as lactoperoxidase and lysozyme, plus some polysaccharides and lipids from animal sources such as chitosan, as well as some fatty acids of animal origin such as eicosatetraenoic acid and docosahexaenoic acid, could also effectively act against Gram-positive and Gram-negative bacteria and fight foodborne microorganisms [219]. Chitosan, as the main part of these groups, was introduced in the previous section. Herein, we introduce some of the other most applicable antimicrobial substances of animal origin.

##### Pleurocidin

Pleurocidin is a novel antimicrobial peptide found in the skin mucous secretions of the winter flounder (*Pseudopleuronectes americanus*). It inhibits the growth of both Gram-positive and Gram-negative foodborne microorganisms. Its effect on some microorganisms, including *Saccharomyces cerevisiae*, *E. coli O157:H7*, *L. monocytogenes*, *Vibrio parahemolyticus*, and *Penicillium expansum*, was confirmed [221] for food preservation purposes. Incorporation of pleurocidin in poly-vinyl alcohol in order to preserve its bioactivity leads to the inhibition of foodborne pathogens, and it shows better efficiency compared to free pleurocidin samples tested with real foods such as apple cider [222].

##### Lactoferrin

Lactoferrin is an 80 kDa glycoprotein containing around 700 amino acids. It is a significant part of the non-heme iron-binding glycoprotein of our organism and belongs to the transferrin class. It is present in most mammalian body fluids such as milk [223]. Anti-inflammatory, antibacterial, antiviral, antioxidant, antitumor, anti-obesity, antibiotic, and immunomodulatory activities are some properties that have been reported to date. Lactoferrin regulates the free iron level in body fluids and causes bacteriostatic properties, which are beneficial for health [224,225].

It exhibits broad-spectrum antimicrobial properties against bacteria, viruses, and fungi [226]. Its bio preservative potential against Salmonella *enterica* and *Escherichia coli O157:H7* as two common dairy pathogens was examined, and it showed a considerable reduction in their growth with only lactoferrin concentrations at or above 112.5 mg/mL in the milk [227]. Lactoferrin’s antimicrobial activity makes it a potential candidate for use in food preservation. Recently, immobilization of lactoferrin in gelatin films improved their optical, mechanical, barrier, and preservative properties [168].

##### Lactoperoxidase

Lactoperoxidase is a heme-containing glycoprotein composed of 608 amino acids with a molecular mass of approximately 78 kDa [228]. It is an antimicrobial enzyme that is secreted in the epithelial cells of the mammary gland and is found in large amounts in cow’s milk [229]. It can destroy bacteria such as *Salmonella*, *Shigella*, *Pseudomonas*, and coliforms. Thereby, it has applications in the food industry for the preservation of unprocessed milk, poultry, fish, and meat [230,231,232,233]. Due to its antimicrobial activity, lactoperoxidase has potential use in food packaging. Its incorporation into defatted soybean meal, a by-product left after crushing soybeans for oil, showed its effectiveness in sliced ham preservation without changing its sensory properties considerably [232].

##### Lysozyme

Lysozyme is another important enzyme widespread throughout the animal and plant kingdoms that shows protective biological functions [234]. Lysozyme is a basic protein in many living organisms, found in body fluids and tissues; therefore, there are many sources of lysozyme [235]. One of the most convenient sources is chicken eggs, the richest in lysozyme. It has interesting characteristics, including antimicrobial activity along with protection against infection, with the effect of strengthening the immune system [236,237]. Despite its interesting properties, the use of free lysozyme in packaging is restricted because it is unstable. Food protease can hydrolyze it and inactivate its catalytic activity [238,239]. The modification of lysozyme via heat, chemicals, and hydrolysis enhances its biological activities and broadens its applications [240]. According to [241], lysozyme coating in packaging films can reduce the water vapor permeability, oxygen permeability, and oil permeability without unwanted effects on the appearance of the films. The effect of lysozyme-N-succinyl chitosan on extending the shelf life of strawberries was proved [242]. Other authors assessed exploiting lysozyme nanofibers as antimicrobial and antioxidant-reinforcing additives by incorporating lysozyme nanofibers into the pullulan matrix as an eco-friendly edible film [243]. Recently, [244] incorporated lysozyme into cold plasma-activated polylactic acid pouches and assessed its effectiveness with pear juice and rice milk-based smoothies. The authors highlighted that activated packages could inhibit *Listeria monocytogenes*. The color of pouches manufactured with this method was also better and more stable [244]. Other studies demonstrated that the synergy of low-dose lysozyme enhanced the inhibition of eugenol–casein nanoparticles against *Staphylococcus aureus* and *Bacillus* sp. by 5.83-fold and 5.53-fold, respectively [245].

#### 3.1.3. Antimicrobial Agent Produced by Microorganisms

Some microorganisms and their derivatives are able to inhibit the growth of other microorganisms [246]. Bacteriocins, produced by some bacteria such as lactic acid bacteria (LAB), are peptides with antimicrobial activity against various non-pathogenic and sometimes pathogenic bacteria [247]. They have recently received much attention due to their antimicrobial activity, especially bacteriocins generated from lactic acid in food and drug systems, most of which are classified as safe agents by the American Food and Drug Administration (FDA) [248].

Current bacteriocins that have the potential for food biopreservation are nisin, leucocin, lactocin 705, enterocin 4, and enterocin produced by various microorganisms [247]. Bacteriocins can be incorporated into packaging films to inhibit spoilage microorganisms and extend the shelf life of foods [247]. The incorporation of bacteriocin from lactic acid bacteria into active food packaging was successful in meat and meat products [249]. The active film obtained from polyvinyl alcohol blended with casein hydrolysates and nisin exhibited antimicrobial activity by reducing the growth of spoilage bacteria and then extending the shelf-life of the packaged food product [250]. The concentrated bacteriocin from lactic acid bacteria was also effective in reducing the growth of *Listeria monocytogenes* in ready-to-eat sliced emulsion-type sausage [251].

Pediocins are bacteriocins produced by *Pediococcus* spp., a group of homofermentative Gram-positive bacteria belonging to the *Lactobacillaceae* family [252]. It is an active peptide that specifically inhibits the growth of *Listeria monocytogenes* [253]. The activity of pediocin can be limited by the presence of salt, heat, or proteolytic enzymes, along with pH and storage time [254]. Pediocin has many applications in the food industry, including the preservation of fermented sausages, vegetables, and dairy products [255]. It can be used directly on food products as a preservative or incorporated into food packaging in order to prevent the spoilage of food products [256]. According to [257], adding nisin and pediocin to starch film inhibited the growth of *L. monocytogenes* and *C. perfringens*.

Reuterin is also an antimicrobial compound produced by *Lactobacillus* spp. strains in the presence of glycerol and is considered an antimicrobial agent against several pathogenic and spoilage microorganisms. Its effectiveness was evaluated for many foodstuffs, including meat, dairy products, beef sausages, and cooked pork, resulting in inhibition of *L. monocytogenes*, *S. aureus*, *E. coli O157:H7*, and so forth. Despite pediocin, reuterins act in a wide range of pH, which makes them a good candidate for biopreservation of many foods. It exhibits broad inhibitory activities against Gram-positive and Gram-negative bacteria, bacterial spores, molds, yeasts, and protozoa [258]. According to [258], reuterin at a concentration of 6.9 mM showed a fungicidal effect in yogurt.

#### 3.1.4. Natural Antimicrobial of Algal and Mushrooms Origin

Mushrooms, with about 140,000 species, have antimicrobial and antioxidant capacities. In addition to their nutritional value, they have preservative and medical properties [259]. Methanol, ethanol, acetone, dichloromethane, and chloroform extracts of many different mushrooms showed inhibitory activity towards pathogenic microorganisms such as *E. coli*, *S. aureus*, *B. subtilis*, *C. albicans*, and *P. aeruginosa* and so forth [246,260]. Antimicrobial and anti-fungal effects of two wild edible mushrooms from the Kashmir valley, *Morchella esculenta* and *Verpa bohemica*, against *E. coli* and *Aspergillus fumigates* were assessed by [261]. Besides, the antimicrobial activity of *Auricularia auricular-judae* mushroom has been confirmed against *Staphylococcus aureus*, *Bacillus subtilis*, *Escherichia coli*, *Pseudomonas aeruginosa*, *Klebsiella pneumoniae*, yeast (*Candida albicans*), and dermatophytic pathogens [262].

Algae also have antimicrobial activity due to their polyphenolic content [263]. Antimicrobial activity of various algae-derived compounds was reported, such as the effectiveness of *Himanthalia elongata*, *Saccharina latissima*, *Laminaria digitate*, *Padina*, and *Dictyota* against *L. monocytogenes*, *Salmonella*, *Enterococcus faecalis*, *P. aeruginosa*, *B. cereus*, and *E. coli* [246]. Attempts to exploit the antimicrobial and antioxidant properties of algae-derived compounds led to their being determined as good candidates for pharmaceutical applications as well as food coatings and films [246]. Polyelectrolyte structured films prepared from cationic starch and sodium alginate showed good thermal, antimicrobial, and surface properties [264].

### 3.2. Antioxidant Active Packaging

Higher than normal concentrations of free radicals during oxidative stress are very harmful to the body as they damage or modify the major components of a cell and cause many diseases. Oxidation is also one of the major causes of food spoilage. Antioxidants are radical scavengers that trap free radicals and help delay or prevent oxidation [265]. Indeed, antioxidants minimize oxygen in the food system by reducing the capacity of active antioxidants. Therefore, they retard both lipid oxidation and protein denaturation. They can also interrupt the oxidation chain reaction and prevent the oxidation reaction along with inactivation of enzyme activity, resulting in blocking catalyzed oxidation reactions [266].

Polyphenols, flavonoids, vitamins C, E, and terpenoids have been reported as natural and synthetic antioxidant agents [267]. Natural antioxidants, compared to synthetic antioxidants, exhibit benefits such as stronger antioxidant effects, better consumer acceptability, potential health benefits, and safety [268].

Two synthetic antioxidants, butylated hydroxyanisole (BHA) and butylated hydroxytoluene (BHT), have been extensively studied in active packaging. However, health awareness and the ever-increasing demand for using natural molecules are encouraging industries to use natural ingredients [269].

In this regard, recently, great consideration has been given to natural antioxidants. Natural antioxidants, also called green antioxidants, are composed of simple phenols, phenolic acids, ascorbic acid, tocopherols, carnosic acid, rutin, carotenoids, flavonoids, vitamins, and anthocyanins [270]. Antioxidant activity is widely observed in fruits, spices, and herbs, such as essential oils [271]. Natural sources of antioxidants comprise blueberries, strawberries, blackberries, rosemary, turmeric, saffron, ginger, chili, green tea, coffee bean, catnip, sage, kenaf seed, roselle seed, thyme, potato peel and sugar beet pulp, spices and herbs, and so forth [270,272,273,274]. The most important group of natural antioxidants are phenolic compounds due to their strong free-radical scavenging effect [266].

Recently, some strategies for incorporating active components such as oxygen scavengers and antioxidants into food packaging have been introduced for food preservation [179]. However, antioxidants may reduce or lose their activities due to interaction with the other ingredients or during processing [26]. The incorporation of antioxidants in the packaging matrix can help to overcome these defects, retard food spoilage, and extend the shelf life of packaged foods [179,275]. Antioxidants applied to food packaging should meet some criteria, such as being low cost, being non-toxic, having high activity at low concentrations, having strong permeability, and showing good stability without affecting the quality of the food [266]. In addition, the proper antioxidants for packaging applications should be chosen based on molecular size, polarity, and release properties of antioxidant compounds [276]. Some forms of antioxidant packaging are already used in the food industry, such as sachets, pads, or labels for foods in which iron and ferrous oxide are common active ingredients [277].

In the past few years, there have been extensive studies on active packaging with antioxidant compounds. A recent study assessed the antioxidant activity of green tea extract for meat preservation. It indicated the potential use of green tea extract in active films [278]. Additionally, polyfunctional starch/tea polyphenol nanofibrous films also demonstrated higher antioxidant activity and optimum mechanical and hydrophobic properties [279]. Moreover, Ref. [280] evaluated the antioxidant activity of chitosan films enriched with *Artemisia campestris* extracts. *Artemisia campestris* is a widespread species of plant in the sunflower family that exhibits antioxidant and UV–Vis barrier properties with good thermal stability. Ref. [281] investigated the influence of *Syzygium cumini* seed extract (SCSE) incorporation in sodium alginate/gum Arabic (SG) films. They highlighted that the addition of SCSE into SG films declined the thermal stability, elongation at break, tensile strength, and moisture content and improved the scavenging activity, opacity, solubility, and water vapor permeability. Ref. [282] produced sodium alginate (SA) maltodextrins (MD) based functional films incorporated with phenolic extract of *Azolla pinnata* leaves fern (AF). They demonstrated that the addition of AF extract to SA.MD films increased the thickness and enhanced the scavenging properties. Furthermore, the film’s solubility, swelling degree, and water vapor permeability were decreased. Ref. [283] fabricated carboxymethyl cellulose (CMC) based novel functional films containing Chinese chives root extract (CRE) and revealed that a higher extract concentration decreased the tensile strength, water solubility, swelling degree, and water vapor permeability. Furthermore, the addition of CRE into CMC exhibited good antioxidant and antimicrobial activity of films.

Antioxidant packaging as an emerging active packaging has great potential for use in food packaging and preserving food in a safe and eco-friendly manner. This technology can consist of biopolymers or nanofillers to improve mechanical or physical properties, resulting in various antioxidant-active packaging for different foodstuffs.

## 4. Active Food Packaging with Nano/Microencapsulated Ingredients

The limitations of using most of these natural antioxidant and antimicrobial agents are their easy degradation, low water solubility, low bioavailability, and undesirable tastes [284]. Encapsulation is a promising technology for overcoming these limitations and enhancing the physical, chemical, and thermal stability along with masking the unwanted taste, increasing the bioavailability and solubility of natural antimicrobial and antioxidant compounds, and providing the possibility of controlled release and targeted delivery [284,285]. Indeed, this innovative technology keeps the sensitive bioactive natural compounds from damage under harsh conditions such as high temperature or pressure, light, oxidation, and neutralization because of unwanted interaction with the other ingredients or foods throughout processing and during storage periods. Thanks to encapsulation techniques, natural antimicrobial and antioxidant compounds are covered by wall material in order to protect their activities against external impacts [286,287].

Encapsulation of antimicrobial agents is highly useful for food applications, notably for active packaging with high breadth potential due to the vast variety of natural antimicrobial compounds and many possible wall materials for encapsulating them. It is a wide search field that has received huge attention recently, specifically the characterization of natural antimicrobial compounds and the choice of compatible shell materials.

**Figure 4:**
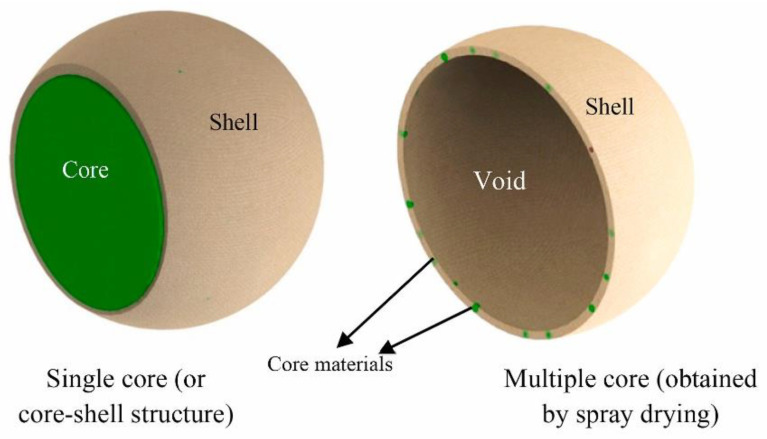
Types of encapsulated particles, in which core materials are natural antimicrobials coated with wall materials as a shell (Reproduced with permission from [285]).

Encapsulation is a process by which one substance in solid, liquid, or gaseous states is entrapped/coated in another material, called wall material. This process can produce different scales of particles, namely, millimeters, micrometers (microencapsulation), and nanometers (nanoencapsulation) [288]. The aim of encapsulation is to produce capsules in nano or micro size. Nano/microcapsules are composed of a core, which is surrounded by a monolithic shell (Figure 4).

The core, which is mainly a bioactive agent, is centered in capsules, and it is also called the internal phase, encapsulant, payload phase, or fill. The shell that coats the core is considered a wall, coating, envelope, membrane, carrier, encapsulating agent, external phase, or matrix [289,290]. The micro/nanocapsules can be classified into four categories depending on whether they have a single- or multi-core and a shell. They can be found in the form of single-core with single-wall, or single-core coated by multi-wall, as well as multi-core with single-wall, or multi-core enveloped by multi-wall [291]. The main outcome of encapsulation is to overcome the physical instability and protect active molecules from degradation in addition to controlling the release of active molecules [292]. Indeed, encapsulation contributes to preserving the active molecule against harsh conditions such as high temperature, pressure, or pH through processing. Moreover, the possibility of controlled release is another important benefit of encapsulation. In this regard, encapsulation could minimize the concentration of active agents needed in different applications by controlling their release and preserving them against harsh conditions.

More specially, the use of encapsulation techniques in the food industry confers several advantages, as mentioned by [291]. Their main advantages are as follows:

- The preservation of sensitive molecules during processing conditions such as phenolic compounds with antimicrobial and antioxidant activity;
- Encapsulation at nano and micro sizes enhances the bioavailability of active molecules;
- The prevention of the alteration of the sensory properties of food by some bioactive agents with unpleasant aroma and taste. Essential oils and oil fish are two examples of active molecules with extreme aromas that can alter the taste of food. Encapsulation prevents the change in taste by covering the molecules and reducing the necessary concentration;
- Controlled release of active compounds to improve food quality and safety;
- The final product of the encapsulation process is mostly a fine powder. It offers several benefits, such as improvement of stability and flowability. It is easier to handle and store the active molecules. In addition, agglomeration and change in density can be reduced by encapsulation.

Different wall materials could be used for the encapsulation, depending on the nature of active molecules. The wall materials should meet some characteristics comprising resistance to high shears and mechanical stresses as well as the ability to incorporate the active molecules with the minimum thickness [293]. In addition, the wall material structures play a key role in encapsulation efficiency and the durability of micro/nanocapsules [294], and their compatibility with the core material is an important element for choosing a wall material. Stronger wall material or a double layer should be chosen for the encapsulation of highly sensitive molecules or under harsh conditions [34]. The apt shell in encapsulation exhibits some criteria, including the stabilization of the core, film-forming ability, flexibility, stability, low density, non-hygroscopicity, the capacity for controlled release, and no unwanted reactions with the payload [285]. Considering all these characteristics, various biopolymers have been applied in the encapsulation of food ingredients [291], including polysaccharides (gum arabic, modified starches, maltodextrins, alginates, pectin, carrageenan, cellulose derivatives, chitosan, and cyclodextrins), fats and waxes (hydrogenated vegetable oils, beeswax, lecithin, medium-chain triglycerides, and glyceryl behenate), proteins (gelatin, whey protein, sodium caseinate, soy protein, gluten, caseins, zein, and silk fibroin), and synthetic compounds (paclitaxel, mPEG500-b-p, polyacrylonitrile, polycaprolactone, polylactic acid, poly methyl methacrylate (PMMA), acrolein, and glycidyl methacrylate epoxy polymers). The natural materials are mostly cheap, abundant, non-toxic, and compatible with food formulations. There are some parameters that should be taken into account for designing the encapsulation process, including capsule size, final physical state, stability, and physical condition of the release [291,295].

The encapsulated particles are divided into three main classes that differ in size, namely, macrocapsules (with a size of more than 5000 µm), microcapsules (0.2–5000 µm), and nanocapsules (with a size of less than 0.2 µm). According to EU regulation, nanoparticles are attributed to the particles when at least 50% of the particles have a size of ≤100 nm [296], whereas pharmaceutical science considers nano size for particles with a size of less than 1000 nm.

Herein, the various techniques of microencapsulation along with nanoencapsulation are mentioned briefly. Microencapsulation techniques include spray drying as a more common technique, spray chilling, spray cooling, extrusion, fluidized bed coating, centrifugal extrusion, freeze-drying, coacervation, interfacial polymerization, molecular inclusion, and so forth [291,295].

**Figure 5:**
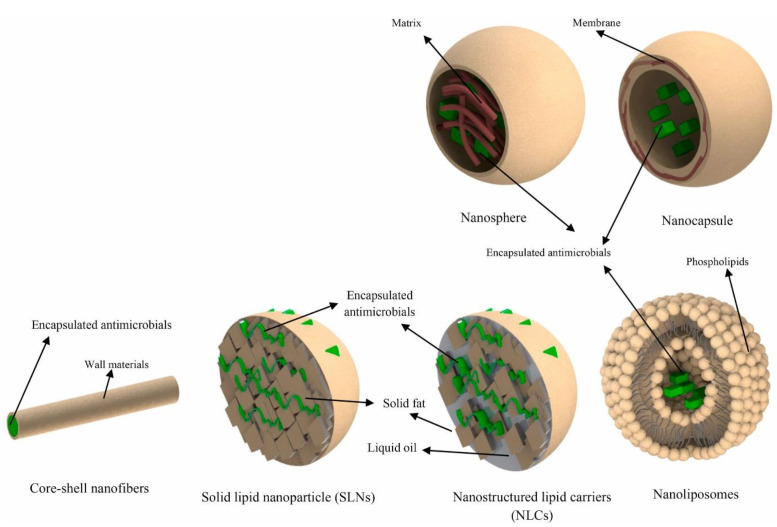
Different nanoencapsulation systems for loading of green natural antimicrobials (Reproduced with permission from [285].

Nanoencapsulation is more recent than microencapsulation. The different nanoencapsulation systems for loading natural antimicrobials are summarized in Figure 5, including nanoemulsions, nanoliposomes, solid lipid nanoparticles (SLNs), nanostructured lipid carriers (NLCs), biopolymeric nanoparticles, and equipment-based nanoencapsulation methods, which have commonly been applied to design the best delivery systems for antimicrobial substances [297]. The selection of the proper technology for each process depends on the physicochemical features, particle size, release type, delivery method, and economic and environmental aspects [291].

In nanoencapsulation, the size of particles is decreased to a nanometer by two basic methods, including top-down (using high-energy devices such as high-pressure-homogenizer, ultrasound, and milling) and bottom-up methods (using low-energy techniques such as spontaneous emulsification or anti-solvent precipitation) [298]. Nanocapsules with a nano-size have a higher ratio of surface to volume. Therefore, they offer higher solubility, bioavailability, and adsorption [34]. Nanoencapsulation exhibits several benefits such as homogeneity, improvement in physical and chemical properties, and shelf stability, in addition to better encapsulation efficiency and boosting the functional properties of active molecules [299]. Besides, the nanocapsules are more controllable. Furthermore, the release of micro-sized particles occurs more slowly, and over longer periods of time compared to nanocapsules. Consequently, a smaller number of nanoparticles is required. Furthermore, according to numerous studies, the emergence of nanotechnology in food packaging, in particular the use of nanocapsules in active packaging, offers interesting benefits such as more efficacy by inhibiting the growth of microorganisms, better thermal protection of bioactive compounds, and better release control [300].

Nanoencapsulation is progressing at a rapid pace in the food sector. The nanoencapsulation of food ingredients aims to produce value-added food products with the possibility of applying this technology in active packaging to boost the preservation of foodstuffs. The incorporation of encapsulated bioactive compounds in the matrix of film packaging is a promising strategy for food preservation, particularly extending the shelf life of fresh foods. Capsules produced through a stable encapsulation process gain remarkable characteristics such as more physiochemical stability and better bioavailability, which show more interest as a delivery system [301,302].

Controlled release packaging (CRP) is a relatively new term that first appeared in the literature in 2005 [303]. A controlled release of bioactive molecules from a film into food is performed during storage periods. The migration of capsules containing antimicrobial or antioxidant compounds can be achieved by direct contact between food and active film packaging or by gas phase diffusion from the packaging layer onto the food surface [304]. The uniqueness of CRP is that it focuses on the kinetics and mechanism of controlled release, what to release, when and how to trigger the release, how much to release, and how fast to release [305]. CRP using antimicrobials may be used for short- or intermediate-term microbial inhibition of highly perishable foods such as fresh meats, seafood, fruit, and vegetables. CRP using antioxidants may be used for long-term retardation of lipid oxidation for shelf-stable foods such as ready-to-eat meals containing fatty components susceptible to oxidation [306].

The control of the release rates of antimicrobial and antimicrobial agents is an emerging area of active food packaging. In fact, rapid release causes consumption of the antimicrobial within a short time, after which the minimum concentration required for the microbial growth inhibition is not maintained. On the other hand, spoilage reactions on the surface may start if the release of the antimicrobial agent from the packaging film is too slow [307].

Different materials and methods may be used in order to apply this strategy by means of various biopolymers and bioactive agents. To date, this technology introduces active antimicrobial packaging with a high potential to minimize the risk of food spoilage and contamination by suppressing the activities of targeted microorganisms. For example, encapsulated essential oils with antimicrobial properties are one of the interesting candidates to be applied for active food packaging. According to [308], lemon myrtle essential oil, encapsulated into cellulose acetate nanofibers, inhibited the growth of 100% *Escherichia coli* and *Staphylococcus aureus* even at the lowest loading concentration of lemon myrtle essential oil of 2 wt%. The antimicrobial activity of cinnamon oil was investigated against foodborne bacteria in free and encapsulated forms, and both exhibit antimicrobial activity. However, the encapsulated form showed antimicrobial action with a lower concentration of 57–125 ppm, compared with non-encapsulated cinnamon, with a concentration of 125–500 ppm. Controlled release of cinnamon capsules leads to better efficiency because the cinnamon concentration in the culture medium reaches its maximum after 4–6 h of incubation when the cells are in the exponential growth phase.

Luteolin, a flavone with potent antioxidant and antimicrobial activities, was encapsulated in oil-in-water nanoemulsions and incorporated into chitosan-matrix [309]. The authors compared films with encapsulated luteolin (CS-LLNEs), films without luteolin (CS-LL), and free luteolin films (CS) as controls. CS-LLNEs film showed slower controlled release to exert antioxidant activity for up to 10 days. In addition, CS-LLNEs film showed better water vapor and oxygen barriers and mechanical properties in comparison with CS-LL film.

Active packaging based on hydroxypropyl methylcellulose containing carvacrol nanoemulsions was developed to extend the shelf-life of wheat bread. The designed system has satisfactory antioxidant activity and good antibacterial activity against *S. aureus* and *E. coli* [310]. Grape seed extract-carvacrol microcapsules were incorporated into chitosan to extend the shelf-life of refrigerated salmon packages. The microcapsules improved the antimicrobial activity of the chitosan film and increased the shelf-life of refrigerated salmon to 4–7 days [311]. The multilayered pectin edible coating was designed with encapsulated *trans*-cinnamaldehyde to extend the shelf life of fresh-cut cantaloupe. Coated fruits lasted longer (7–9 days) compared to uncoated controls (4 days) at 4 °C [312].

Eventually, biodegradable film packaging containing capsules has great potential to meet the needs of three main goals of developed packaging science, comprising sustainability, safety, and convenience [31,313]. Recent progress in the incorporation of nano/microencapsulated natural agents into active food packaging systems is summarized in Table 3.

## 5. Conclusions and Future Developments

In recent years, sustainable packaging has been adopted by the food industry, either as a repackaging of environmental policies, as a marketing strategy in response to social pressures, or as a genuine attempt to grapple with the commercial, social, and environmental issues associated with plastic packaging. This review paper summarizes the current state of biodegradable and active packaging biopolymers as well as major antioxidant and antimicrobial natural agents incorporated into the polymers as nano-microcapsules. More than 300 sources were reviewed, including scientific literature and commercial information.

In conclusion, this review shows that biodegradable active biopolymers are generally characterized by low water vapor barrier properties; hence, the modification of their structure or other techniques can be convenient for modulating this property. These efforts can lead to the production of biodegradable active biopolymers with good barrier and mechanical properties. At the same time, more efforts should be implemented to decrease their price and their competition with food by increasing the use of second- and third-generation feedstock. Another challenge is the lack of a collection channel for these bioplastics. Further studies are necessary to establish biodegradable monolayer or multilayer materials as a viable alternative to commonly used fossil fuel-based materials for food products. A techno-economic analysis (TEA) combined with a life cycle assessment (LCA) should be implemented to estimate and quantify costs, emissions, and energy intensity associated with material acquisition, processing, transport, and end-of-life treatment of biodegradable packaging production.

At the same time, nano-microencapsulation of natural antimicrobial and antioxidant agents improves their physical, chemical, and thermal stability along with increasing their bioavailability and solubility, which provides the possibility of their controlled release and targeted delivery. However, this review highlighted that strict European regulations for food contact materials and safety aspects restricted the application of many encapsulated antimicrobial and antioxidant agents in the releasing systems. Eliminating legislative restrictions and creating a unique global organization would allow for more specific and exact legislation for food-contact materials. Meanwhile, the use of nanotechnology in the antimicrobial and antioxidant food packaging films requires the control and monitoring of nanomaterials as well as risk assessment, which requires information about their toxicity and exposure [296]. In line with this, the European Food Safety Authority (EFSA) has recently produced guidance for the risk assessment of nanoscience and nanotechnology applications in the food and feed chain [320]. Moreover, an effort should be made to familiarize the public consumer with active food packaging and increase their acceptance of such an innovation. This review contributes to increasing the knowledge of the available sustainable biodegradable active packaging and highlights the increasing number of investigations and the increasing industrial interest in the area, as well as further developments needed.

## Figures and Tables

**Table 1:** Overview of major commercial biodegradable polymers.

Biodegradable Polymers	Commercial Name	Company
Polyhydroxyalkanoate (PHA)/Polyhydroxybutyrate (PHB)	Minerv	Bio-On, Italy
	Biocycle	PHB Industrial, Brazil
	Biomer	Biomer, Germany
	Nodax	Danimer Scientific, USA,
	AmBio	Shenzhen Ecomann, China
	Kaneka	Kaneka Corporation, Japan
	Solon	RWDC Industries, Singapore
	ENMAT	TianAn Biologic Mat., China
	Hydal	Bochemie, Czech Republic
	Green Bio	Tianjin Green-Bio, China
	PHB	Imperial Chemical Industries, UK
	TephaFLEX	TEPHA, USA
	ENMAT	Tinam, China
	PHA	SIRIM, Malaysia
Starch	Solanyl	Rodenburg, Netherlands
	BiomeHT	Biome Bioplastics, UK
	Starch	Green Home, South Africa
	MATER-BI	Novamont, Italy
	Starch	Biobag, Norway
	Cardia	Cardia Bioplastics, Australia
	Starch	Starch Tech Inc., USA
	Starch	Evercorn, Japan
Casein/Whey proteins	Casein	Lactips, France
	Wheylayer	Wheylayer ltd, Germany
polybutylene succinate (PBS)	PBSA Bionolle	Highpolymer, Japan
	EnPol, PBSA	Ire chemicals, South Korea
	PBSA	Kingfa, China
	PBSA	IPC-CAS, China
Polybutylene adipate terephthalate (PBAT)	Ecoflex	BASF, Germany
	Biomax	Dupont, USA
	MATER-BI	Novamont, Italy
	Easter Bio	Eastman Chemicals, USA
Cellulose	CNF Eco, Cartocan	Toppan, Japan
	MelOx	Klabin, Brazil
	Cellulose	International paper, USA
	NatureFlex	Futamura, Japan
	TIPA	TIPA Corp, Israel
	Zelfo	The Green Factory, France
	Microcel	Roquette, France
Poly(lactic acid)	PLA	Bio4pack, Germany
	PLA INGEO	NatureWorks, USA
	CPLA	Great River, China
	PLA	Galactic, Belgium
	L-PLA	Corbion, Netherlands
	Bio-Flex	FKuR, Germany
	NATIVIA	Taghleef Industries, UAE
	PLA	Minima Technology, Taiwan
	PLA	Naturabiomat, Austria
	PLA	Natur-Tec, USA
	Ecovio	BASF, Germany

**Table 3:** Incorporation of nano/microencapsulated natural agents in active food packaging systems.

Packaging Material and Encapsulated Antimicrobial System	Purpose	References
Active packaging based on hydroxypropyl methylcellulose containing carvacrol nanoemulsions	Development of active packaging system to extend the shelf life of wheat bread. The designed system has a satisfactory antioxidant activity, good antibacterial activity against *S. aureus* and *E. coli*.	[310]
Edible coating fabricated with chitosan, pectin, and encapsulated *trans*-cinnamaldehyde	Designing a multilayered edible coating with antimicrobial agents to extend the shelf life of fresh-cut cantaloupe stored at 4 °C	[312]
Alginate coating containing nano-emulsified basil oil	Development of a coating system against the following spoilage fungi: *Penicillium chrysogenum* and *Aspergillus flavus*	[314]
Active packaging based on hydroxypropyl methylcellulose containing oregano essential oil nanoemulsions	Higher antimicrobial activity against all tested bacterial strains, particularly *S. typhimurium*	[315]
Starch-carboxy methyl cellulose films containing rosemary essential oil (REO)-loaded benzoic acid-chitosan (BA-CS) nanogel	Using of encapsulated REO into BA-CS nanogel in film structure to obtain immediately (REO) and gradual (nanogel) antimicrobial effect against *S. aureus*	[316]
Polylactide films containing essential oils/nanoparticles	Inhibiting the growth of *L. monocytogenes* and *S. typhimurium* on contaminated cheese	[10]
Active packaging containing cinnamon-loaded nanophytosomes into electrospun nanofiber	Higher antimicrobial activity and improving the shelf life of shrimp	[317]
Active packaging based on cellulose nanocrystals (CNCs) reinforced chitosan, containing thyme-oregano, thyme-tea tree, and thyme-peppermint nanoemulsions	Development of active antifungal packaging for rice preservation. Chitosan-based nanocomposite films loaded essential oils mixtures showed significant antifungal activity against *Aspergillus niger*, *Aspergillus flavus*, *Aspergillus parasiticus*, and *Penicillium chrysogenum*, reducing their growth by 51–77%.	[318]
Encapsulation of gallic acid into lentil flour-based nanofibers by electrospinning technology and use of these nanofibers as active packaging materials	Enhancement of the oxidative stability of walnuts present in active packages with encapsulated gallic. The reduction in oxidation of walnuts with lower peroxide, *p*-anisidine, and TOTOX values was observed.	[319]
Active packaging film based on chitosan with grape seed extract-carvacrol microcapsules	Development of active film to extend the shelf-life of refrigerated salmon. The microcapsules improved the antimicrobial activity of the chitosan film and increased the shelf-life of refrigerated salmon to 4–7 days.	[311]

## References

[1] Bahrami A., Delshadi R., Assadpour E., Jafari S.M., Williams L. (2020). Antimicrobial-loaded nanocarriers for food packaging applications. Adv. Colloid Interface Sci..

[2] Chawla R., Sivakumar S., Kaur H. (2021). Antimicrobial edible films in food packaging: Current scenario and recent nanotechnological advancements—A review. Carbohydr. Polym. Technol. Appl..

[3] Sharma S., Barkauskaite S., Jaiswal A.K., Jaiswal S. (2021). Essential oils as additives in active food packaging. Food Chem..

[4] Ibarra G.V., Sendón R., Rodríguez-Bernaldo de Quirós A., Barros-Velázquez J. (2016). Chapter 29—Antimicrobial Food Packaging Based on Biodegradable Materials. Antimicrobial Food Packaging.

[5] López-Carballo G., Gómez-Estaca J., Catalá R., Hernández-Muñoz P., Gavara R., Yam K.L., Lee D.S. (2012). 3-Active antimicrobial food and beverage packaging. Emerging Food Packaging Technologies.

[6] Ahamed A., Veksha A., Giannis A., Lisak G. (2021). Flexible packaging plastic waste-environmental implications, management solutions, and the way forward. Curr. Opin. Chem. Eng..

[7] Kunwar B., Cheng H.N., Chandrashekaran S.R., Sharma B.K. (2016). Plastics to fuel: A review. Renew. Sustain. Energy Rev..

[8] Alojaly H., Benyounis K.Y. (2020). Packaging With Plastics and Polymeric Materials. Reference Module in Materials Science and Materials Engineering.

[9] Zhang W., Zhang Y., Cao J., Jiang W. (2021). Improving the performance of edible food packaging films by using nanocellulose as an additive. Int. J. Biol. Macromol..

[10] Ahmed J., Hiremath N., Jacob H. (2017). Antimicrobial efficacies of essential oils/nanoparticles incorporated polylactide films against *L. monocytogenes* and *S. typhimurium* on contaminated cheese. Int. J. Food Prop..

[11] Artham T., Doble M. (2008). Biodegradation of Aliphatic and Aromatic Polycarbonates. Macromol. Biosci..

[12] Geyer R., Jambeck J.R., Law K.L. (2017). Production, use, and fate of all plastics ever made. Sci. Adv..

[13] Mohamed S.A.A., El-Sakhawy M., El-Sakhawy M.A.-M. (2020). Polysaccharides, Protein and Lipid -Based Natural Edible Films in Food Packaging: A Review. Carbohydr. Polym..

[14] Pereira J.M., Rodríguez Y., Blasco-Monleon S., Porter A., Lewis C., Pham C.K. (2020). Microplastic in the stomachs of open-ocean and deep-sea fishes of the North-East Atlantic. Environ. Pollut..

[15] Roman L., Kastury F., Petit S., Aleman R., Wilcox C., Hardesty B.D., Hindell M.A. (2020). Plastic, nutrition and pollution; relationships between ingested plastic and metal concentrations in the livers of two Pachyptila seabirds. Sci. Rep..

[16] Mattsson K., Johnson E.V., Malmendal A., Linse S., Hansson L.-A., Cedervall T. (2017). Brain damage and behavioural disorders in fish induced by plastic nanoparticles delivered through the food chain. Sci. Rep..

[17] Ali S.S., Elsamahy T., Al-Tohamy R., Zhu D., Mahmoud Y.A.-G., Koutra E., Metwally M.A., Kornaros M., Sun J. (2021). Plastic wastes biodegradation: Mechanisms, challenges and future prospects. Sci. Total Environ..

[18] Kershaw P. (2015). Sources, Fate and Effects of Microplastics in the Marine Environment: A Global Assessment.

[19] Abioye O.P., Abioye A.A., Afolalu S.A., Ongbali S.O. (2018). A Review of Biodegradable Plastics in Nigeria. Int. J. Mech. Eng. Technol. IJMET.

[20] Hopewell J., Dvorak R., Kosior E. (2009). Plastics recycling: Challenges and opportunities. Philos. Trans. R. Soc. B Biol. Sci..

[21] Rai P., Mehrotra S., Priya S., Gnansounou E., Sharma S.K. (2021). Recent advances in the sustainable design and applications of biodegradable polymers. Bioresour. Technol..

[22] Udayakumar G.P., Muthusamy S., Selvaganesh B., Sivarajasekar N., Rambabu K., Banat F., Sivamani S., Sivakumar N., Hosseini-Bandegharaei A., Show P.L. (2021). Biopolymers and composites: Properties, characterization and their applications in food, medical and pharmaceutical industries. J. Environ. Chem. Eng..

[23] Zhong Y., Godwin P., Jin Y., Xiao H. (2020). Biodegradable polymers and green-based antimicrobial packaging materials: A mini-review. Adv. Ind. Eng. Polym. Res..

[24] Jeevahan J., Chandrasekaran M. (2019). Nanoedible films for food packaging: A review. J. Mater. Sci..

[25] Reichert C.L., Bugnicourt E., Coltelli M.-B., Cinelli P., Lazzeri A., Canesi I., Braca F., Martínez B.M., Alonso R., Agostinis L. (2020). Bio-Based Packaging: Materials, Modifications, Industrial Applications and Sustainability. Polymers.

[26] Mastromatteo M., Mastromatteo M., Conte A., Del Nobile M.A. (2010). Advances in controlled release devices for food packaging applications. Trends Food Sci. Technol..

[27] Jideani V.A., Vogt K. (2016). Antimicrobial Packaging for Extending the Shelf Life of Bread-A Review. Crit. Rev. Food Sci. Nutr..

[28] Khaneghah M.A., Hashemi S.M.B., Eş I., Fracassetti D., Limbo S. (2018). Efficacy of Antimicrobial Agents for Food Contact Applications: Biological Activity, Incorporation into Packaging, and Assessment Methods: A Review. J. Food Prot..

[29] Firouz S.M., Mohi-Alden K., Omid M. (2021). A critical review on intelligent and active packaging in the food industry: Research and development. Food Res. Int..

[30] Khaneghah M.A., Hashemi S.M.B., Limbo S. (2018). Antimicrobial agents and packaging systems in antimicrobial active food packaging: An overview of approaches and interactions. Food Bioprod. Process..

[31] Ribeiro-Santos R., Andrade M., Sanches-Silva A. (2017). Application of encapsulated essential oils as antimicrobial agents in food packaging. Curr. Opin. Food Sci..

[32] Unalan I., Boccaccini A.R. (2021). Essential oils in biomedical applications: Recent progress and future opportunities. Curr. Opin. Biomed. Eng..

[33] Dhifi W., Bellili S., Jazi S., Bahloul N., Mnif W. (2016). Essential Oils’ Chemical Characterization and Investigation of Some Biological Activities: A Critical Review. Medicines.

[34] Delshadi R., Bahrami A., Tafti A.G., Barba F.J., Williams L.L. (2020). Micro and nano-encapsulation of vegetable and essential oils to develop functional food products with improved nutritional profiles. Trends Food Sci. Technol..

[35] Gómez B., Barba F.J., Domínguez R., Putnik P., Bursać Kovačević D., Pateiro M., Toldrá F., Lorenzo J.M. (2018). Microencapsulation of antioxidant compounds through innovative technologies and its specific application in meat processing. Trends Food Sci. Technol..

[36] Jurić S., Jurić M., Siddique M.A.B., Fathi M. (2020). Vegetable Oils Rich in Polyunsaturated Fatty Acids: Nanoencapsulation Methods and Stability Enhancement. Food Rev. Int..

[37] Alehosseini E., Jafari S.M. (2019). Micro/nano-encapsulated phase change materials (PCMs) as emerging materials for the food industry. Trends Food Sci. Technol..

[38] Rochman C.M., Browne M.A., Halpern B.S., Hentschel B.T., Hoh E., Karapanagioti H.K., Rios-Mendoza L.M., Takada H., Teh S., Thompson R.C. (2013). Classify plastic waste as hazardous. Nature.

[39] Andrady A.L. (2011). Microplastics in the marine environment. Mar. Pollut. Bull..

[40] Webb H.K., Arnott J., Crawford R.J., Ivanova E.P. (2013). Plastic Degradation and Its Environmental Implications with Special Reference to Poly(ethylene terephthalate). Polymers.

[41] (2000). EN 13432 Packaging—Requirements for Packaging Recoverable through Composting and Biodegradation—Test Scheme and Evaluation Criteria for the Final Acceptance of Packaging.

[42] Ali S.S., Elsamahy T., Koutra E., Kornaros M., El-Sheekh M., Abdelkarim E.A., Zhu D., Sun J. (2021). Degradation of conventional plastic wastes in the environment: A review on current status of knowledge and future perspectives of disposal. Sci. Total Environ..

[43] Gorrasi G., Sorrentino A., Lichtfouse E. (2021). Back to plastic pollution in COVID times. Environ. Chem. Lett..

[44] Horodytska O., Cabanes A., Fullana A. (2019). Plastic Waste Management: Current Status and Weaknesses. The Handbook of Environmental Chemistry.

[45] Helinski O.K., Poor C.J., Wolfand J.M. (2021). Ridding our rivers of plastic: A framework for plastic pollution capture device selection. Mar. Pollut. Bull..

[46] Hamilton: Solving Plastic Pollution through Accountability. https://scholar.google.com/scholar_lookup?title=Solving%20Plastic%20Pollution%20Through%20Accountability&publication_year=2019&author=A.%20Hamilton&author=R.%20Scheer&author=T.%20Stakes&author=S.%20Allan.

[47] Xu H., Sheng J., Wu X., Zhan K., Tao S., Wen X., Liu W., Cudjoe O., Tao F. (2021). Moderating effects of plastic packaged food on association of urinary phthalate metabolites with emotional symptoms in Chinese adolescents. Ecotoxicol. Environ. Saf..

[48] Larrain M., Van Passel S., Thomassen G., Van Gorp B., Nhu T.T., Huysveld S., Van Geem K.M., De Meester S., Billen P. (2021). Techno-economic assessment of mechanical recycling of challenging post-consumer plastic packaging waste. Resour. Conserv. Recycl..

[49] Liu C., Zhang X., Medda F. (2021). Plastic credit: A consortium blockchain-based plastic recyclability system. Waste Manag..

[50] Mattila H., Virtanen T., Vartiainen T., Ruuskanen J. (1992). Emissions of polychlorinated dibenzo-p-dioxins and dibenzofurans in flue gas from co-combustion of mixed plastics with coal and bark. Chemosphere.

[51] Ahmadzadeh S., Khaneghah M.A. (2020). Role of Green Polymers in Food Packaging. Encycl. Renew. Sustain. Mater..

[52] Lindström T., Österberg F. (2020). Evolution of biobased and nanotechnology packaging—A review. Nord. Pulp Pap. Res. J..

[53] EUBIO_Admin Market. Eur. Bioplastics E.V..

[54] Hawthorne L.M., Beganović A., Schwarz M., Noordanus A.W., Prem M., Zapf L., Scheibel S., Margreiter G., Huck C.W., Bach K. (2020). Suitability of Biodegradable Materials in Comparison with Conventional Packaging Materials for the Storage of Fresh Pork Products over Extended Shelf-Life Periods. Foods.

[55] Ivanković A., Zeljko K., Talić S., Bevanda A.M., Lasić M. (2017). Biodegradable Packaging in The Food Industry. Arch. Für Leb..

[56] Kobayashi S. (2010). Lipase-catalyzed polyester synthesis—A green polymer chemistry. Proc. Jpn. Acad. Ser. B.

[57] Van den Oever M., Molenveld K., Zee M., Bos H. (2017). Bio-Based and Biodegradable Plastics—Facts and Figures. Focus on Food Packaging in The Netherlands.

[58] Ghuttora N. Increase the Usage of Biopolymers and Biodegradable Polymers for Sustainable Environment. http://www.theseus.fi/handle/10024/121984.

[59] Öner E.T., Fang Z. (2013). Microbial Production of Extracellular Polysaccharides from Biomass. Pretreatment Techniques for Biofuels and Biorefineries.

[60] Shevkani K., Singh N., Bajaj R., Kaur A. (2017). Wheat starch production, structure, functionality and applications—A review. Int. J. Food Sci. Technol..

[61] Deng N., Deng Z., Tang C., Liu C., Luo S., Chen T., Hu X. (2021). Formation, structure and properties of the starch-polyphenol inclusion complex: A review. Trends Food Sci. Technol..

[62] Oyeyinka S.A., Akintayo O.A., Adebo O.A., Kayitesi E., Njobeh P.B. (2021). A review on the physicochemical properties of starches modified by microwave alone and in combination with other methods. Int. J. Biol. Macromol..

[63] Żołek-Tryznowska Z., Holica J. (2020). Starch films as an environmentally friendly packaging material: Printing performance. J. Clean. Prod..

[64] Cheng H., Chen L., McClements D.J., Yang T., Zhang Z., Ren F., Miao M., Tian Y., Jin Z. (2021). Starch-based biodegradable packaging materials: A review of their preparation, characterization and diverse applications in the food industry. Trends Food Sci. Technol..

[65] Mali S., Grossmann M.V.E., Garcia M.A., Martino M.N., Zaritzky N.E. (2002). Microstructural characterization of yam starch films. Carbohydr. Polym..

[66] Nawab A., Alam F., Haq M.A., Lutfi Z., Hasnain A. (2017). Mango kernel starch-gum composite films: Physical, mechanical and barrier properties. Int. J. Biol. Macromol..

[67] Nordin N., Othman S.H., Rashid S.A., Basha R.K. (2020). Effects of glycerol and thymol on physical, mechanical, and thermal properties of corn starch films. Food Hydrocoll..

[68] Adamus J., Spychaj T., Zdanowicz M., Jędrzejewski R. (2018). Thermoplastic starch with deep eutectic solvents and montmorillonite as a base for composite materials. Ind. Crops Prod..

[69] Thirmizir M.Z.A., Ishak Z.A.M., Salim M.S., Gutiérrez T.J. (2020). Compatibilization and Crosslinking in Biodegradable Thermoplastic Polyester Blends. Reactive and Functional Polymers Volume Two: Modification Reactions, Compatibility and Blends.

[70] Nafchi A.M., Alias A.K., Mahmud S., Robal M. (2012). Antimicrobial, rheological, and physicochemical properties of sago starch films filled with nanorod-rich zinc oxide. J. Food Eng..

[71] Vaezi K., Asadpour G., Sharifi H. (2019). Effect of ZnO nanoparticles on the mechanical, barrier and optical properties of thermoplastic cationic starch/montmorillonite biodegradable films. Int. J. Biol. Macromol..

[72] Ali A., Xie F., Yu L., Liu H., Meng L., Khalid S., Chen L. (2018). Preparation and characterization of starch-based composite films reinfoced by polysaccharide-based crystals. Compos. Part B Eng..

[73] de Souza A.C., Dias A.M.A., Sousa H.C., Tadini C.C. (2014). Impregnation of cinnamaldehyde into cassava starch biocomposite films using supercritical fluid technology for the development of food active packaging. Carbohydr. Polym..

[74] Avila-Sosa R., Palou E., Jiménez Munguía M.T., Nevárez-Moorillón G.V., Navarro Cruz A.R., López-Malo A. (2012). Antifungal activity by vapor contact of essential oils added to amaranth, chitosan, or starch edible films. Int. J. Food Microbiol..

[75] Souza A.C., Goto G.E.O., Mainardi J.A., Coelho A.C.V., Tadini C.C. (2013). Cassava starch composite films incorporated with cinnamon essential oil: Antimicrobial activity, microstructure, mechanical and barrier properties. LWT-Food Sci. Technol..

[76] Engel J.B., Ambrosi A., Tessaro I.C. (2019). Development of biodegradable starch-based foams incorporated with grape stalks for food packaging. Carbohydr. Polym..

[77] Sganzerla W.G., Rosa G.B., Ferreira A.L.A., da Rosa C.G., Beling P.C., Xavier L.O., Hansen C.M., Ferrareze J.P., Nunes M.R., Barreto P.L.M. (2020). Bioactive food packaging based on starch, citric pectin and functionalized with Acca sellowiana waste by-product: Characterization and application in the postharvest conservation of apple. Int. J. Biol. Macromol..

[78] Baek S.-K., Kim S., Song K.B. (2019). Cowpea starch films containing maqui berry extract and their application in salmon packaging. Food Packag. Shelf Life.

[79] Cheng J., Wang H., Kang S., Xia L., Jiang S., Chen M., Jiang S. (2019). An active packaging film based on yam starch with eugenol and its application for pork preservation. Food Hydrocoll..

[80] Zhou X., Yang R., Wang B., Chen K. (2019). Development and characterization of bilayer films based on pea starch/polylactic acid and use in the cherry tomatoes packaging. Carbohydr. Polym..

[81] Hassan M., Dave K., Chandrawati R., Dehghani F., Gomes V.G. (2019). 3D printing of biopolymer nanocomposites for tissue engineering: Nanomaterials, processing and structure-function relation. Eur. Polym. J..

[82] Panrong T., Karbowiak T., Harnkarnsujarit N. (2019). Thermoplastic starch and green tea blends with LLDPE films for active packaging of meat and oil-based products. Food Packag. Shelf Life.

[83] Moreno O., Atarés L., Chiralt A., Cruz-Romero M.C., Kerry J. (2018). Starch-Gelatin Antimicrobial Packaging Materials to Extend the Shelf Life of Chicken Breast Fillets.

[84] Fadeyibi A., Osunde Z.D., Egwim E.C., Idah P.A. (2017). Performance evaluation of cassava starch-zinc nanocomposite film for tomatoes packaging. J. Agric. Eng..

[85] Hu L., Du H., Liu C., Zhang Y., Yu G., Zhang X., Si C., Li B., Peng H. (2019). Comparative Evaluation of the Efficient Conversion of Corn Husk Filament and Corn Husk Powder to Valuable Materials via a Sustainable and Clean Biorefinery Process. ACS Sustain. Chem. Eng..

[86] Liu W., Du H., Liu H., Xie H., Xu T., Zhao X., Liu Y., Zhang X., Si C. (2020). Highly Efficient and Sustainable Preparation of Carboxylic and Thermostable Cellulose Nanocrystals via FeCl3-Catalyzed Innocuous Citric Acid Hydrolysis. ACS Sustain. Chem. Eng..

[87] Zhao G., Du J., Chen W., Pan M., Chen D. (2019). Preparation and thermostability of cellulose nanocrystals and nanofibrils from two sources of biomass: Rice straw and poplar wood. Cellulose.

[88] Liu Y., Ahmed S., Sameen D.E., Wang Y., Lu R., Dai J., Li S., Qin W. (2021). A review of cellulose and its derivatives in biopolymer-based for food packaging application. Trends Food Sci. Technol..

[89] Pooresmaeil M., Behzadi Nia S., Namazi H. (2019). Green encapsulation of LDH(Zn/Al)-5-Fu with carboxymethyl cellulose biopolymer; new nanovehicle for oral colorectal cancer treatment. Int. J. Biol. Macromol..

[90] Cazón P., Vázquez M. (2021). Bacterial cellulose as a biodegradable food packaging material: A review. Food Hydrocoll..

[91] Wang W., Gu F., Deng Z., Zhu Y., Zhu J., Guo T., Song J., Xiao H. (2021). Multilayer surface construction for enhancing barrier properties of cellulose-based packaging. Carbohydr. Polym..

[92] Assis R.Q., Pagno C.H., Stoll L., Rios P.D., Rios A.d.O., Olivera F.C. (2021). Active food packaging of cellulose acetate: Storage stability, protective effect on oxidation of riboflavin and release in food simulants. Food Chem..

[93] Blanco-Cabra N., Paetzold B., Ferrar T., Mazzolini R., Torrents E., Serrano L., LLuch-Senar M. (2020). Characterization of different alginate lyases for dissolving Pseudomonas aeruginosa biofilms. Sci. Rep..

[94] Günter E.A., Popeyko O.V., Belozerov V.S., Martinson E.A., Litvinets S.G. (2020). Physicochemical and swelling properties of composite gel microparticles based on alginate and callus cultures pectins with low and high degrees of methylesterification. Int. J. Biol. Macromol..

[95] Lee K.Y., Mooney D.J. (2012). Alginate: Properties and biomedical applications. Prog. Polym. Sci..

[96] Xu M., Qin M., Cheng Y., Niu X., Kong J., Zhang X., Huang D., Wang H. (2021). Alginate microgels as delivery vehicles for cell-based therapies in tissue engineering and regenerative medicine. Carbohydr. Polym..

[97] Parreidt S.T., Müller K., Schmid M. (2018). Alginate-Based Edible Films and Coatings for Food Packaging Applications. Foods.

[98] Zhang H., Zhang F., Yuan R., Chen Y. (2020). Chapter 13—Applications of natural polymer-based hydrogels in the food industry. Hydrogels Based on Natural Polymers.

[99] Abdel Aziz M.S., Salama H.E. (2018). Effect of vinyl montmorillonite on the physical, responsive and antimicrobial properties of the optimized polyacrylic acid/chitosan superabsorbent via Box-Behnken model. Int. J. Biol. Macromol..

[100] Aziz M.S.A., Salama H.E., Saad G.R. (2019). Diglycidyl ether of bisphenol A/chitosan-graft-polyaniline composites with electromagnetic interference shielding properties: Synthesis, characterization, and curing kinetics. Polym. Eng. Sci..

[101] Salama H.E., Aziz A.M.S. (2021). Development of active edible coating of alginate and aloe vera enriched with frankincense oil for retarding the senescence of green capsicums. LWT.

[102] Bennacef C., Desobry-Banon S., Probst L., Desobry S. (2021). Advances on alginate use for spherification to encapsulate biomolecules. Food Hydrocoll..

[103] Chen J., Wu A., Yang M., Ge Y., Pristijono P., Li J., Xu B., Mi H. (2021). Characterization of sodium alginate-based films incorporated with thymol for fresh-cut apple packaging. Food Control.

[104] Dong Y., Wei Z., Xue C. (2021). Recent advances in carrageenan-based delivery systems for bioactive ingredients: A review. Trends Food Sci. Technol..

[105] Khotimchenko M., Tiasto V., Kalitnik A., Begun M., Khotimchenko R., Leonteva E., Bryukhovetskiy I., Khotimchenko Y. (2020). Antitumor potential of carrageenans from marine red algae. Carbohydr. Polym..

[106] Zia K.M., Tabasum S., Nasif M., Sultan N., Aslam N., Noreen A., Zuber M. (2017). A review on synthesis, properties and applications of natural polymer based carrageenan blends and composites. Int. J. Biol. Macromol..

[107] Liu Y., Qin Y., Bai R., Zhang X., Yuan L., Liu J. (2019). Preparation of pH-sensitive and antioxidant packaging films based on κ-carrageenan and mulberry polyphenolic extract. Int. J. Biol. Macromol..

[108] Pourashouri P., Shabanpour B., Heydari S., Raeisi S. (2021). Encapsulation of fish oil by carrageenan and gum tragacanth as wall materials and its application to the enrichment of chicken nuggets. LWT.

[109] Priyadarshi R., Rhim J.-W. (2020). Chitosan-based biodegradable functional films for food packaging applications. Innov. Food Sci. Emerg. Technol..

[110] Yu J., Wang D., Geetha N., Khawar K.M., Jogaiah S., Mujtaba M. (2021). Current trends and challenges in the synthesis and applications of chitosan-based nanocomposites for plants: A review. Carbohydr. Polym..

[111] Mujtaba M., Morsi R.E., Kerch G., Elsabee M.Z., Kaya M., Labidi J., Khawar K.M. (2019). Current advancements in chitosan-based film production for food technology; A review. Int. J. Biol. Macromol..

[112] Wang H., Ding F., Ma L., Zhang Y. (2021). Edible films from chitosan-gelatin: Physical properties and food packaging application. Food Biosci..

[113] Wang H., Qian J., Ding F. (2018). Emerging Chitosan-Based Films for Food Packaging Applications. J. Agric. Food Chem..

[114] Alirezalu K., Pirouzi S., Yaghoubi M., Karimi-Dehkordi M., Jafarzadeh S., Mousavi Khaneghah A. (2021). Packaging of beef fillet with active chitosan film incorporated with ɛ-polylysine: An assessment of quality indices and shelf life. Meat Sci..

[115] Xavier L.O., Sganzerla W.G., Rosa G.B., da Rosa C.G., Agostinetto L., de Lima Veeck A.P., Bretanha L.C., Micke G.A., Dalla Costa M., Bertoldi F.C. (2021). Chitosan packaging functionalized with Cinnamodendron dinisii essential oil loaded zein: A proposal for meat conservation. Int. J. Biol. Macromol..

[116] Dubey N.K., Dubey R., Pal K., Banerjee I., Sarkar P., Kim D., Deng W.-P., Dubey N.K., Majumder K. (2020). Chapter 27—Edible films and coatings: An update on recent advances. Biopolymer-Based Formulations.

[117] Tang X.Z., Kumar P., Alavi S., Sandeep K.P. (2012). Recent Advances in Biopolymers and Biopolymer-Based Nanocomposites for Food Packaging Materials. Crit. Rev. Food Sci. Nutr..

[118] Zhang H., Mittal G. (2010). Biodegradable protein-based films from plant resources: A review. Environ. Prog. Sustain. Energy.

[119] Ciannamea E.M., Stefani P.M., Ruseckaite R.A. (2016). Properties and antioxidant activity of soy protein concentrate films incorporated with red grape extract processed by casting and compression molding. LWT.

[120] Eswaranandam S., Hettiarachchy N.S., Johnson M.G. (2004). Antimicrobial Activity of Citric, Lactic, Malic, or Tartaric Acids and Nisin-incorporated Soy Protein Film Against Listeria monocytogenes, *Escherichia coli* O157:H7, and *Salmonella gaminara*. J. Food Sci..

[121] Soares R.M.D., Scremin F.F., Soldi V. (2005). Thermal Stability of Biodegradable Films Based on Soy Protein and Corn Starch. Macromol. Symp..

[122] Su J.-F., Huang Z., Liu K., Fu L.-L., Liu H.-R. (2007). Mechanical Properties, Biodegradation and Water Vapor Permeability of Blend Films of Soy Protein Isolate and Poly (vinyl alcohol) Compatibilized by Glycerol. Polym. Bull..

[123] Tian H., Guo G., Fu X., Yao Y., Yuan L., Xiang A. (2018). Fabrication, properties and applications of soy-protein-based materials: A review. Int. J. Biol. Macromol..

[124] Rhim J.-W., Lee J.H., Ng P.K.W. (2007). Mechanical and barrier properties of biodegradable soy protein isolate-based films coated with polylactic acid. LWT-Food Sci. Technol..

[125] Xiao Y., Liu Y., Kang S., Xu H. (2021). Insight into the formation mechanism of soy protein isolate films improved by cellulose nanocrystals. Food Chem..

[126] Li J., Li X., Zhang F., Zhang W., Li J. (2021). Facile design of tough, strong, and UV-shielding soy protein-based composite films. Ind. Crops Prod..

[127] Jansens K.J.A., Vo Hong N., Telen L., Brijs K., Lagrain B., Van Vuure A.W., Van Acker K., Verpoest I., Van Puyvelde P., Goderis B. (2013). Effect of molding conditions and moisture content on the mechanical properties of compression molded glassy, wheat gluten bioplastics. Ind. Crops Prod..

[128] Wang J., Wei Z., Li L., Bian K., Zhao M. (2009). Characteristics of enzymatic hydrolysis of thermal-treated wheat gluten. J. Cereal Sci..

[129] Tunc S., Angellier H., Cahyana Y., Chalier P., Gontard N., Gastaldi E. (2007). Functional properties of wheat gluten/montmorillonite nanocomposite films processed by casting. J. Membr. Sci..

[130] Rovera C., Türe H., Hedenqvist M.S., Farris S. (2020). Water vapor barrier properties of wheat gluten/silica hybrid coatings on paperboard for food packaging applications. Food Packag. Shelf Life.

[131] Gutiérrez T.J., Mendieta J.R., Ortega-Toro R. (2021). In-depth study from gluten/PCL-based food packaging films obtained under reactive extrusion conditions using chrome octanoate as a potential food grade catalyst. Food Hydrocoll..

[132] Türe H., Gällstedt M., Hedenqvist M.S. (2012). Antimicrobial compression-moulded wheat gluten films containing potassium sorbate. Food Res. Int..

[133] Mascheroni E., Guillard V., Gastaldi E., Gontard N., Chalier P. (2011). Anti-microbial effectiveness of relative humidity-controlled carvacrol release from wheat gluten/montmorillonite coated papers. Food Control.

[134] Zhang X., Dong C., Hu Y., Gao M., Luan G. (2021). Zein as a structural protein in gluten-free systems: An overview. Food Sci. Hum. Wellness.

[135] Shukla R., Cheryan M. (2001). Zein: The industrial protein from corn. Ind. Crops Prod..

[136] Zhou L., Wang Y. (2021). Physical and antimicrobial properties of zein and methyl cellulose composite films with plasticizers of oleic acid and polyethylene glycol. LWT.

[137] Lai H.-M., Padua G.W. (1997). Properties and Microstructure of Plasticized Zein Films. Cereal Chem..

[138] Wang Y., Rakotonirainy A.M., Padua G.W. (2003). Thermal Behavior of Zein-based Biodegradable Films. Starch -Stärke.

[139] Ghanbarzadeh B., Oromiehi A.R. (2008). Biodegradable biocomposite films based on whey protein and zein: Barrier, mechanical properties and AFM analysis. Int. J. Biol. Macromol..

[140] Huo W., Wei D., Zhu W., Li Z., Jiang Y. (2018). High-elongation zein films for flexible packaging by synergistic plasticization: Preparation, structure and properties. J. Cereal Sci..

[141] Xu H., Zhang G. (2012). Synergistic Effect of Oleic Acid and Glycerol on Zein Film Plasticization. J. Agric. Food Chem..

[142] Kashiri M., López-Carballo G., Hernández-Muñoz P., Gavara R. (2019). Antimicrobial packaging based on a LAE containing zein coating to control foodborne pathogens in chicken soup. Int. J. Food Microbiol..

[143] Ahammed S., Liu F., Wu J., Khin M.N., Yokoyama W.H., Zhong F. (2021). Effect of transglutaminase crosslinking on solubility property and mechanical strength of gelatin-zein composite films. Food Hydrocoll..

[144] Daniloski D., Petkoska A.T., Lee N.A., Bekhit A.E.-D., Carne A., Vaskoska R., Vasiljevic T. (2021). Active edible packaging based on milk proteins: A route to carry and deliver nutraceuticals. Trends Food Sci. Technol..

[145] Horne D.S., Boland M., Singh H. (2020). Chapter 6—Casein micelle structure and stability. Milk Proteins.

[146] Fitzsimons S.M., Mulvihill D.M., Morris E.R. (2007). Denaturation and aggregation processes in thermal gelation of whey proteins resolved by differential scanning calorimetry. Food Hydrocoll..

[147] Boland M., Boland M., Singh H. (2020). Chapter 21—Milk proteins: The future. Milk Proteins.

[148] Martins J.T., Bourbon A.I., Pinheiro A.C., Fasolin L.H., Vicente A.A. (2018). Protein-Based Structures for Food Applications: From Macro to Nanoscale. Front. Sustain. Food Syst..

[149] Moughan P.J., Boland M., Singh H. (2020). Chapter 17—Milk proteins: A rich source of bioactives for developing functional foods. Milk Proteins.

[150] Bonilla J., Paiano R.B., Lourenço R.V., Bittante A.M.Q.B., Sobral P.J.A. (2021). Biodegradation of Films Based on Natural and Synthetic Biopolymers Using an Aquatic System from Active Sludge. J. Polym. Environ..

[151] Aguirre-Joya J.A., De Leon-Zapata M.A., Alvarez-Perez O.B., Torres-León C., Nieto-Oropeza D.E., Ventura-Sobrevilla J.M., Aguilar M.A., Ruelas-Chacón X., Rojas R., Ramos-Aguiñaga M.E., Grumezescu A.M., Holban A.M. (2018). Chapter 1—Basic and Applied Concepts of Edible Packaging for Foods. Food Packaging and Preservation.

[152] Makhijani K., Kumar R., Sharma S.K. (2015). Biodegradability of Blended Polymers: A Comparison of Various Properties. Crit. Rev. Environ. Sci. Technol..

[153] Tkaczewska J. (2020). Peptides and protein hydrolysates as food preservatives and bioactive components of edible films and coatings—A review. Trends Food Sci. Technol..

[154] Ponce A., Roura S.I., Moreira M.R., Barros-Velázquez J. (2016). Chapter 37—Casein and Chitosan Polymers: Use in Antimicrobial Packaging. Antimicrobial Food Packaging.

[155] Haque E., Chand R., Kapila S. (2008). Biofunctional Properties of Bioactive Peptides of Milk Origin. Food Rev. Int..

[156] Zhao Q., Shi Y., Wang X., Huang A. (2020). Characterization of a novel antimicrobial peptide from buffalo casein hydrolysate based on live bacteria adsorption. J. Dairy Sci..

[157] Bhosale S., Fulpagare Y., Desale R. (2019). International Journal of Advanced Research in Biological Sciences Nanoliposomes: Applications in Food and Dairy Industry. Int. J. Adv. Res. Biol. Sci..

[158] Broumand A., Emam-Djomeh Z., Hamedi M., Razavi S.H. (2011). Antimicrobial, water vapour permeability, mechanical and thermal properties of casein based Zataraia multiflora Boiss. Extract containing film. LWT-Food Sci. Technol..

[159] Dinika I., Verma D.K., Balia R., Utama G.L., Patel A.R. (2020). Potential of cheese whey bioactive proteins and peptides in the development of antimicrobial edible film composite: A review of recent trends. Trends Food Sci. Technol..

[160] Uranga J., Leceta I., Etxabide A., Guerrero P., de la Caba K. (2016). Cross-linking of fish gelatins to develop sustainable films with enhanced properties. Eur. Polym. J..

[161] Weng W., Zheng H. (2015). Effect of transglutaminase on properties of tilapia scale gelatin films incorporated with soy protein isolate. Food Chem..

[162] Gómez-Guillén M.C., Giménez B., López-Caballero M.E., Montero M.P. (2011). Functional and bioactive properties of collagen and gelatin from alternative sources: A review. Food Hydrocoll..

[163] Gómez-Guillén M.C., Pérez-Mateos M., Gómez-Estaca J., López-Caballero E., Giménez B., Montero P. (2009). Fish gelatin: A renewable material for developing active biodegradable films. Trends Food Sci. Technol..

[164] Suderman N., Isa M.I.N., Sarbon N.M. (2018). The effect of plasticizers on the functional properties of biodegradable gelatin-based film: A review. Food Biosci..

[165] Limpisophon K., Tanaka M., Osako K. (2010). Characterisation of gelatin–fatty acid emulsion films based on blue shark (Prionace glauca) skin gelatin. Food Chem..

[166] Jiménez A., Fabra M.J., Talens P., Chiralt A. (2012). Effect of re-crystallization on tensile, optical and water vapour barrier properties of corn starch films containing fatty acids. Food Hydrocoll..

[167] Roy S., Rhim J.-W. (2020). Preparation of antimicrobial and antioxidant gelatin/curcumin composite films for active food packaging application. Colloids Surf. B Biointerfaces.

[168] Tavassoli M., Sani M.A., Khezerlou A., Ehsani A., McClements D.J. (2021). Multifunctional nanocomposite active packaging materials: Immobilization of quercetin, lactoferrin, and chitosan nanofiber particles in gelatin films. Food Hydrocoll..

[169] Khwaldia K., Arab-Tehrany E., Desobry S. (2010). Biopolymer Coatings on Paper Packaging Materials. Compr. Rev. Food Sci. Food Saf..

[170] Akoh C.C. (2017). Food Lipids: Chemistry, Nutrition, and Biotechnology.

[171] Azar N.F.A., Pezeshki A., Ghanbarzadeh B., Hamishehkar H., Mohammadi M. (2020). Nanostructured lipid carriers: Promising delivery systems for encapsulation of food ingredients. J. Agric. Food Res..

[172] Costa-Fernandez S., Mattos J.K.R., Scheunemann G.S., Salata G.C., Chorilli M., Watanabe I.-S., de Araujo G.L.B., Santos M.F., Ishida K., Lopes L.B. (2021). Nanostructured lipid carriers containing chitosan or sodium alginate for co-encapsulation of antioxidants and an antimicrobial agent for potential application in wound healing. Int. J. Biol. Macromol..

[173] Syahida N.S., Ismail-Fitry M.R., Ainun Z.M.A., Nur Hanani Z.A. (2020). Effects of palm wax on the physical, mechanical and water barrier properties of fish gelatin films for food packaging application. Food Packag. Shelf Life.

[174] de Oliveira Filho J.G., Bezerra C.C.D.O.N., Albiero B.R., Oldoni F.C.A., Miranda M., Egea M.B., de Azeredo H.M.C., Ferreira M.D. (2020). New approach in the development of edible films: The use of carnauba wax micro- or nanoemulsions in arrowroot starch-based films. Food Packag. Shelf Life.

[175] National Academies of Sciences, Engineering, and Medicine (2016). Genetically Engineered Crops: Experiences and Prospects.

[176] Baldevraj R.S.M., Jagadish R.S., Lagarón J.-M. (2011). 14-Incorporation of chemical antimicrobial agents into polymeric films for food packaging. Multifunctional and Nanoreinforced Polymers for Food Packaging.

[177] Sung S.-Y., Sin L.T., Tee T.-T., Bee S.-T., Rahmat A.R., Rahman W.A.W.A., Tan A.-C., Vikhraman M. (2013). Antimicrobial agents for food packaging applications. Trends Food Sci. Technol..

[178] Ibrahim S., Elsayed H., Hasanin M. (2021). Biodegradable, Antimicrobial and Antioxidant Biofilm for Active Packaging Based on Extracted Gelatin and Lignocelluloses Biowastes. J. Polym. Environ..

[179] Kuai L., Liu F., Chiou B.-S., Avena-Bustillos R.J., McHugh T.H., Zhong F. (2021). Controlled release of antioxidants from active food packaging: A Review. Food Hydrocoll..

[180] Kruijf N.D., Beest M.V., Rijk R., Sipiläinen-Malm T., Losada P.P., Meulenaer B.D. (2002). Active and intelligent packaging: Applications and regulatory aspects. Food Addit. Contam..

[181] Dainelli D., Baughan J.S. (2015). 8-Global legislation for active and intelligent packaging materials. Global Legislation for Food Contact Materials.

[182] Mendonca A., Jackson-Davis A., Moutiq R., Thomas-Popo E., Ricke S.C., Atungulu G.G., Rainwater C.E., Park S.H. (2018). Chapter 14—Use of Natural Antimicrobials of Plant Origin to Improve the Microbiological Safety of Foods. Food and Feed Safety Systems and Analysis.

[183] Göksen G., Fabra M.J., Ekiz H.I., López-Rubio A. (2020). Phytochemical-loaded electrospun nanofibers as novel active edible films: Characterization and antibacterial efficiency in cheese slices. Food Control.

[184] Lin L., Gu Y., Cui H. (2019). Moringa oil/chitosan nanoparticles embedded gelatin nanofibers for food packaging against Listeria monocytogenes and Staphylococcus aureus on cheese. Food Packag. Shelf Life.

[185] Meira S.M.M., Zehetmeyer G., Scheibel J.M., Werner J.O., Brandelli A. (2016). Starch-halloysite nanocomposites containing nisin: Characterization and inhibition of Listeria monocytogenes in soft cheese. LWT-Food Sci. Technol..

[186] Cui H.Y., Wu J., Li C.Z., Lin L. (2016). Anti-listeria effects of chitosan-coated nisin-silica liposome on Cheddar cheese. J. Dairy Sci..

[187] Soto K.M., Hernández-Iturriaga M., Loarca-Piña G., Luna-Bárcenas G., Mendoza S. (2019). Antimicrobial effect of nisin electrospun amaranth: Pullulan nanofibers in apple juice and fresh cheese. Int. J. Food Microbiol..

[188] Cui H., Bai M., Li C., Liu R., Lin L. (2018). Fabrication of chitosan nanofibers containing tea tree oil liposomes against Salmonella spp. in chicken. LWT.

[189] Woraprayote W., Pumpuang L., Tosukhowong A., Zendo T., Sonomoto K., Benjakul S., Visessanguan W. (2018). Antimicrobial biodegradable food packaging impregnated with Bacteriocin 7293 for control of pathogenic bacteria in pangasius fish fillets. LWT.

[190] Vishnu Priya N., Vinitha U.G., Meenakshi Sundaram M. (2021). Preparation of chitosan-based antimicrobial active food packaging film incorporated with Plectranthus amboinicus essential oil. Biocatal. Agric. Biotechnol..

[191] Kanatt S.R. (2020). Development of active/intelligent food packaging film containing Amaranthus leaf extract for shelf life extension of chicken/fish during chilled storage. Food Packag. Shelf Life.

[192] Meral R., Alav A., Karakas C., Dertli E., Yilmaz M.T., Ceylan Z. (2019). Effect of electrospun nisin and curcumin loaded nanomats on the microbial quality, hardness and sensory characteristics of rainbow trout fillet. LWT.

[193] Khah M.D., Ghanbarzadeh B., Roufegarinejad Nezhad L., Ostadrahimi A. (2021). Effects of virgin olive oil and grape seed oil on physicochemical and antimicrobial properties of pectin-gelatin blend emulsified films. Int. J. Biol. Macromol..

[194] Dhumal C.V., Ahmed J., Bandara N., Sarkar P. (2019). Improvement of antimicrobial activity of sago starch/guar gum bi-phasic edible films by incorporating carvacrol and citral. Food Packag. Shelf Life.

[195] Göksen G., Fabra M.J., Pérez-Cataluña A., Ekiz H.I., Sanchez G., López-Rubio A. (2021). Biodegradable active food packaging structures based on hybrid cross-linked electrospun polyvinyl alcohol fibers containing essential oils and their application in the preservation of chicken breast fillets. Food Packag. Shelf Life.

[196] Alizadeh-Sani M., Mohammadian E., McClements D.J. (2020). Eco-friendly active packaging consisting of nanostructured biopolymer matrix reinforced with TiO_2_ and essential oil: Application for preservation of refrigerated meat. Food Chem..

[197] Montero Y., Souza A.G., Oliveira É.R., dos Santos Rosa D. (2021). Nanocellulose functionalized with cinnamon essential oil: A potential application in active biodegradable packaging for strawberry. Sustain. Mater. Technol..

[198] He X., Li M., Gong X., Niu B., Li W. (2021). Biodegradable and antimicrobial CSC films containing cinnamon essential oil for preservation applications. Food Packag. Shelf Life.

[199] Cui H., Bai M., Rashed M.M.A., Lin L. (2018). The antibacterial activity of clove oil/chitosan nanoparticles embedded gelatin nanofibers against Escherichia coli O157:H7 biofilms on cucumber. Int. J. Food Microbiol..

[200] Min T., Sun X., Yuan Z., Zhou L., Jiao X., Zha J., Zhu Z., Wen Y. (2021). Novel antimicrobial packaging film based on porous poly(lactic acid) nanofiber and polymeric coating for humidity-controlled release of thyme essential oil. LWT.

[201] Thielmann J., Theobald M., Wutz A., Krolo T., Buergy A., Niederhofer J., Welle F., Muranyi P. (2021). Litsea cubeba fruit essential oil and its major constituent citral as volatile agents in an antimicrobial packaging material. Food Microbiol..

[202] Tao R., Sedman J., Ismail A. (2021). Antimicrobial activity of various essential oils and their application in active packaging of frozen vegetable products. Food Chem..

[203] Kaur M., Santhiya D. (2021). UV-shielding antimicrobial zein films blended with essential oils for active food packaging. J. Appl. Polym. Sci..

[204] Raeisi M., Mohammadi M.A., Coban O.E., Ramezani S., Ghorbani M., Tabibiazar M., Khoshbakht R., Noori S.M.A. (2021). Physicochemical and antibacterial effect of Soy Protein Isolate/Gelatin electrospun nanofibres incorporated with Zataria multiflora and Cinnamon zeylanicum essential oils. J. Food Meas. Charact..

[205] Jamróz E., Juszczak L., Kucharek M. (2018). Investigation of the physical properties, antioxidant and antimicrobial activity of ternary potato starch-furcellaran-gelatin films incorporated with lavender essential oil. Int. J. Biol. Macromol..

[206] Akhter R., Masoodi F.A., Wani T.A., Rather S.A. (2019). Functional characterization of biopolymer based composite film: Incorporation of natural essential oils and antimicrobial agents. Int. J. Biol. Macromol..

[207] Abdollahi M., Rezaei M., Farzi G. (2012). Improvement of active chitosan film properties with rosemary essential oil for food packaging. Int. J. Food Sci. Technol..

[208] Yeddes W., Djebali K., Aidi Wannes W., Horchani-Naifer K., Hammami M., Younes I., Saidani Tounsi M. (2020). Gelatin-chitosan-pectin films incorporated with rosemary essential oil: Optimized formulation using mixture design and response surface methodology. Int. J. Biol. Macromol..

[209] Bahrami A., Fattahi R. (2021). Biodegradable carboxymethyl cellulose–polyvinyl alcohol composite incorporated with Glycyrrhiza Glabra L. essential oil: Physicochemical and antibacterial features. Food Sci. Nutr..

[210] Asbahani A.E., Miladi K., Badri W., Sala M., Addi E.H.A., Casabianca H., Mousadik A.E., Hartmann D., Jilale A., Renaud F.N.R. (2015). Essential oils: From extraction to encapsulation. Int. J. Pharm..

[211] Elshafie H.S., Camele I. (2017). An Overview of the Biological Effects of Some Mediterranean Essential Oils on Human Health. BioMed Res. Int..

[212] Tariq S., Wani S., Rasool W., Shafi K., Bhat M.A., Prabhakar A., Shalla A.H., Rather M.A. (2019). A comprehensive review of the antibacterial, antifungal and antiviral potential of essential oils and their chemical constituents against drug-resistant microbial pathogens. Microb. Pathog..

[213] Guttierrez J., Rodriguez G., Barry-Ryan C., Bourke P. (2008). Efficacy of Plant Essential Oils against Foodborne Pathogens and Spoilage Bacteria Associated with Ready-to-Eat Vegetables: Antimicrobial and Sensory Screening. J. Food Prot..

[214] Dušan F., Marián S., Katarína D., Dobroslava B. (2006). Essential oils—Their antimicrobial activity against Escherichia coli and effect on intestinal cell viability. Toxicol. Vitro.

[215] Fernández-López J., Viuda-Martos M. (2018). Introduction to the Special Issue: Application of Essential Oils in Food Systems. Foods.

[216] Zhu Y., Li C., Cui H., Lin L. (2021). Encapsulation strategies to enhance the antibacterial properties of essential oils in food system. Food Control.

[217] Rolim H.M.L., Ramalho T.C., Rai M., dos Santos C.A. (2021). Chapter 7—Biopolymer essential oil nanocomposite for antimicrobial packaging. Biopolymer-Based Nano Films.

[218] Priyadarshi R., Sauraj, Kumar B., Deeba F., Kulshreshtha A., Negi Y.S. (2018). Chitosan films incorporated with Apricot (Prunus armeniaca) kernel essential oil as active food packaging material. Food Hydrocoll..

[219] Pisoschi A.M., Pop A., Georgescu C., Turcuş V., Olah N.K., Mathe E. (2018). An overview of natural antimicrobials role in food. Eur. J. Med. Chem..

[220] Hoskin D.W., Ramamoorthy A. (2008). Studies on anticancer activities of antimicrobial peptides. Biochim. Biophys. Acta BBA-Biomembr..

[221] Burrowes O.J., Hadjicharalambous C., Diamond G., Lee T.-C. (2004). Evaluation of Antimicrobial Spectrum and Cytotoxic Activity of Pleurocidin for Food Applications. J. Food Sci..

[222] Wang X., Yue T., Lee T. (2015). Development of Pleurocidin-poly(vinyl alcohol) electrospun antimicrobial nanofibers to retain antimicrobial activity in food system application. Food Control.

[223] Antoshin A.A., Shpichka A.I., Huang G., Chen K., Lu P., Svistunov A.A., Lychagin A.V., Lipina M.M., Sinelnikov M.Y., Reshetov I.V. (2021). Lactoferrin as a regenerative agent: The old-new panacea?. Pharmacol. Res..

[224] Siqueiros-Cendón T., Arévalo-Gallegos S., Iglesias-Figueroa B.F., García-Montoya I.A., Salazar-Martínez J., Rascón-Cruz Q. (2014). Immunomodulatory effects of lactoferrin. Acta Pharmacol. Sin..

[225] Niaz B., Saeed F., Ahmed A., Imran M., Maan A.A., Khan M.K.I., Tufail T., Anjum F.M., Hussain S., Suleria H.A.R. (2019). Lactoferrin (LF): A natural antimicrobial protein. Int. J. Food Prop..

[226] Krupińska A.M., Bogucki Z. (2021). Clinical aspects of the use of lactoferrin in dentistry. J. Oral Biosci..

[227] Biernbaum E.N., Gnezda A., Akbar S., Franklin R., Venturelli P.A., McKillip J.L. (2021). Lactoferrin as an antimicrobial against Salmonella enterica and Escherichia coli O157:H7 in raw milk. JDS Commun..

[228] Boots J.-W., Floris R. (2006). Lactoperoxidase: From catalytic mechanism to practical applications. Int. Dairy J..

[229] Saeed F., Afzaal M., Tufail T., Ahmad A. (2019). Use of Natural Antimicrobial Agents: A Safe Preservation Approach. Active Antimicrobial Food Packaging.

[230] Armenteros M., Dalvit P., Leyva V., Ponce P., Alfonso P. (2007). Risk analysis of the exacerbation of foodborne pathogens in raw milk activated with the lactoperoxidase system. Rev. Salud Anim..

[231] Jooyandeh H., Aberoumand A., Nasehi B. (2011). Application of Lactoperoxidase System in Fish and Food Products: A Review. Am. Eurasian J. Agric. Environ. Sci..

[232] Seifu E., Buys E.M., Donkin E.F. (2005). Significance of the lactoperoxidase system in the dairy industry and its potential applications: A review. Trends Food Sci. Technol..

[233] Munsch-Alatossava P., Gursoy O., Lorilla P.M., Gauchi J.-P., Alatossava T., Holban A.M., Grumezescu A.M. (2018). Chapter 15—Antibacterial Effects and Modes of Action of the Activated Lactoperoxidase System (LPS), of CO_2_ and N_2_ Gas as Food-Grade Approaches to Control Bovine Raw Milk–Associated Bacteria. Food Control and Biosecurity.

[234] Leśnierowski G., Yang T. (2021). Lysozyme and its modified forms: A critical appraisal of selected properties and potential. Trends Food Sci. Technol..

[235] Wu T., Jiang Q., Wu D., Hu Y., Chen S., Ding T., Ye X., Liu D., Chen J. (2019). What is new in lysozyme research and its application in food industry? A review. Food Chem..

[236] Khan M.I., Dowarha D., Katte R., Chou R.-H., Filipek A., Yu C. (2019). Lysozyme as the anti-proliferative agent to block the interaction between S100A6 and the RAGE V domain. PLoS ONE.

[237] Ragland S.A., Criss A.K. (2017). From bacterial killing to immune modulation: Recent insights into the functions of lysozyme. PLoS Pathog..

[238] Zhou X., Deng J., Fang C., Yu R., Lei W., He X., Zhang C. (2020). Preparation and characterization of lysozyme@carbon nanotubes/waterborne polyurethane composite and the potential application in printing inks. Prog. Org. Coat..

[239] Huang W., Li X., Xue Y., Huang R., Deng H., Ma Z. (2013). Antibacterial multilayer films fabricated by LBL immobilizing lysozyme and HTCC on nanofibrous mats. Int. J. Biol. Macromol..

[240] Carrillo W., Spindola H., Ramos M., Recio I., Carvalho J.E. (2016). Anti-Inflammatory and Anti-Nociceptive Activities of Native and Modified Hen Egg White Lysozyme. J. Med. Food.

[241] Zhang Z., Zhou X., Wang D., Fang C., Zhang W., Wang C., Huang Z. (2021). Lysozyme-based composite membranes and their potential application for active packaging. Food Biosci..

[242] Niu X., Zhu L., Xi L., Guo L., Wang H. (2020). An antimicrobial agent prepared by N-succinyl chitosan immobilized lysozyme and its application in strawberry preservation. Food Control.

[243] Silva N.H.C.S., Vilela C., Almeida A., Marrucho I.M., Freire C.S.R. (2018). Pullulan-based nanocomposite films for functional food packaging: Exploiting lysozyme nanofibers as antibacterial and antioxidant reinforcing additives. Food Hydrocoll..

[244] Glicerina V., Siroli L., Canali G., Chinnici F., Capelli F., Lanciotti R., Colombo V., Romani S. (2021). Efficacy of biodegradable, antimicrobial packaging on safety and quality parameters maintenance of a pear juice and rice milk-based smoothie product. Food Control.

[245] Wang Y., Xue Y., Bi Q., Qin D., Du Q., Jin P. (2021). Enhanced antibacterial activity of eugenol-entrapped casein nanoparticles amended with lysozyme against gram-positive pathogens. Food Chem..

[246] Quinto E.J., Caro I., Villalobos-Delgado L.H., Mateo J., De-Mateo-Silleras B., Redondo-Del-Río M.P. (2019). Food Safety through Natural Antimicrobials. Antibiotics.

[247] Daba G.M., Elkhateeb W.A. (2020). Bacteriocins of lactic acid bacteria as biotechnological tools in food and pharmaceuticals: Current applications and future prospects. Biocatal. Agric. Biotechnol..

[248] Agriopoulou S., Stamatelopoulou E., Sachadyn-Król M., Varzakas T. (2020). Lactic Acid Bacteria as Antibacterial Agents to Extend the Shelf Life of Fresh and Minimally Processed Fruits and Vegetables: Quality and Safety Aspects. Microorganisms.

[249] Woraprayote W., Malila Y., Sorapukdee S., Swetwiwathana A., Benjakul S., Visessanguan W. (2016). Bacteriocins from lactic acid bacteria and their applications in meat and meat products. Meat Sci..

[250] Settier-Ramírez L., López-Carballo G., Gavara R., Hernández-Muñoz P. (2021). Broadening the antimicrobial spectrum of nisin-producing Lactococcus lactis subsp. Lactis to Gram-negative bacteria by means of active packaging. Int. J. Food Microbiol..

[251] Bungenstock L., Abdulmawjood A., Reich F. (2021). Suitability of lactic acid bacteria and deriving antibacterial preparations to enhance shelf-life and consumer safety of emulsion type sausages. Food Microbiol..

[252] Haakensen M., Dobson C.M., Hill J.E., Ziola B. (2021). 2009 Reclassification of Pediococcus dextrinicus (Coster and White 1964) Back 1978 (Approved Lists 1980) as Lactobacillus dextrinicus comb. nov., and emended description of the genus Lactobacillus. Int. J. Syst. Evol. Microbiol..

[253] Gabrielsen C., Brede D.A., Nes I.F., Diep D.B. (2014). Circular bacteriocins: Biosynthesis and mode of action. Appl. Environ. Microbiol..

[254] Niamah A.K. (2018). Structure, mode of action and application of pediocin natural antimicrobial food preservative: A review. Basrah J Agric Sci.

[255] Verma S.K., Sood S.K., Saini R.K., Saini N. (2017). Pediocin PA-1 containing fermented cheese whey reduces total viable count of raw buffalo (*Bubalis bubalus*) milk. LWT-Food Sci. Technol..

[256] Espitia P.J.P., Otoni C.G., Soares N.F.F., Barros-Velázquez J. (2016). Chapter 36—Pediocin Applications in Antimicrobial Food Packaging Systems. Antimicrobial Food Packaging.

[257] Meira S.M.M., Zehetmeyer G., Werner J.O., Brandelli A. (2017). A novel active packaging material based on starch-halloysite nanocomposites incorporating antimicrobial peptides. Food Hydrocoll..

[258] Vimont A., Fernandez B., Ahmed G., Fortin H.-P., Fliss I. (2019). Quantitative antifungal activity of reuterin against food isolates of yeasts and moulds and its potential application in yogurt. Int. J. Food Microbiol..

[259] Bal C. (2018). Benefits and Uses of Mushroom. J. Bacteriol. Mycol. Open Access.

[260] El-Saber Batiha G., Hussein D.E., Algammal A.M., George T.T., Jeandet P., Al-Snafi A.E., Tiwari A., Pagnossa J.P., Lima C.M., Thorat N.D. (2021). Application of natural antimicrobials in food preservation: Recent views. Food Control.

[261] Shameem N., Kamili A.N., Ahmad M., Masoodi F.A., Parray J.A. (2017). Antimicrobial activity of crude fractions and morel compounds from wild edible mushrooms of North western Himalaya. Microb. Pathog..

[262] Oli A.N., Edeh P.A., Al-Mosawi R.M., Mbachu N.A., Al-Dahmoshi H.O.M., Al-Khafaji N.S.K., Ekuma U.O., Okezie U.M., Saki M. (2020). Evaluation of the phytoconstituents of Auricularia auricula-judae mushroom and antimicrobial activity of its protein extract. Eur. J. Integr. Med..

[263] Devi K.P., Suganthy N., Kesika P., Pandian S.K. (2008). Bioprotective properties of seaweeds: In vitro evaluation of antioxidant activity and antimicrobial activity against food borne bacteria in relation to polyphenolic content. BMC Complement. Altern. Med..

[264] Şen F., Uzunsoy İ., Baştürk E., Kahraman M.V. (2017). Antimicrobial agent-free hybrid cationic starch/sodium alginate polyelectrolyte films for food packaging materials. Carbohydr. Polym..

[265] Chand K., Rajeshwari, Hiremathad A., Singh M., Santos M.A., Keri R.S. (2017). A review on antioxidant potential of bioactive heterocycle benzofuran: Natural and synthetic derivatives. Pharmacol. Rep..

[266] Choe E., Min D.B. (2009). Mechanisms of Antioxidants in the Oxidation of Foods. Compr. Rev. Food Sci. Food Saf..

[267] Fritz K.L., Seppanen C.M., Kurzer M.S., Saari Csallany A. (2003). The in vivo antioxidant activity of soybean isoflavones in human subjects. Nutr. Res..

[268] Bera D., Lahiri D., Nag A. (2006). Studies on a natural antioxidant for stabilization of edible oil and comparison with synthetic antioxidants. J. Food Eng..

[269] Jamshidian M., Tehrany E.A., Desobry S. (2012). Release of synthetic phenolic antioxidants from extruded poly lactic acid (PLA) film. Food Control.

[270] Bodoira R.M., Penci M.C., Ribotta P.D., Martínez M.L. (2017). Chia (*Salvia hispanica* L.) oil stability: Study of the effect of natural antioxidants. LWT.

[271] Jiang J., Xiong Y.L. (2016). Natural antioxidants as food and feed additives to promote health benefits and quality of meat products: A review. Meat Sci..

[272] Pandey A.K., Kumar P., Singh P., Tripathi N.N., Bajpai V.K. (2017). Essential Oils: Sources of Antimicrobials and Food Preservatives. Front. Microbiol..

[273] Baştürk A., Ceylan M.M., Çavuş M., Boran G., Javidipour I. (2018). Effects of some herbal extracts on oxidative stability of corn oil under accelerated oxidation conditions in comparison with some commonly used antioxidants. LWT.

[274] Celano R., Piccinelli A.L., Pagano I., Roscigno G., Campone L., De Falco E., Russo M., Rastrelli L. (2017). Oil distillation wastewaters from aromatic herbs as new natural source of antioxidant compounds. Food Res. Int..

[275] Dong Z., Xu F., Ahmed I., Li Z., Lin H. (2018). Characterization and preservation performance of active polyethylene films containing rosemary and cinnamon essential oils for Pacific white shrimp packaging. Food Control.

[276] Nerin C., Silva F., Manso S., Becerril R., Barros-Velázquez J. (2016). Chapter 6—The Downside of Antimicrobial Packaging: Migration of Packaging Elements into Food. Antimicrobial Food Packaging.

[277] Mills A. (2005). Oxygen indicators and intelligent inks for packaging food. Chem. Soc. Rev..

[278] Song X.-C., Canellas E., Wrona M., Becerril R., Nerin C. (2020). Comparison of two antioxidant packaging based on rosemary oleoresin and green tea extract coated on polyethylene terephthalate for extending the shelf life of minced pork meat. Food Packag. Shelf Life.

[279] Zhang D., Chen L., Cai J., Dong Q., Din Z., Hu Z.-Z., Wang G.-Z., Ding W.-P., He J.-R., Cheng S.-Y. (2021). Starch/tea polyphenols nanofibrous films for food packaging application: From facile construction to enhance mechanical, antioxidant and hydrophobic properties. Food Chem..

[280] Moalla S., Ammar I., Fauconnier M.-L., Danthine S., Blecker C., Besbes S., Attia H. (2021). Development and characterization of chitosan films carrying *Artemisia campestris* antioxidants for potential use as active food packaging materials. Int. J. Biol. Macromol..

[281] Abdin M., El-Beltagy A.E., El-sayed M.E., Naeem M.A. (2021). Production and Characterization of Sodium Alginate/Gum Arabic Based Films Enriched with Syzygium cumini Seeds Extracts for Food Application. J. Polym. Environ..

[282] Eltabakh M., Kassab H., Badawy W., Abdin M., Abdelhady S. (2021). Active Bio-composite Sodium Alginate/Maltodextrin Packaging Films for Food Containing Azolla pinnata Leaves Extract as Natural Antioxidant. J. Polym. Environ..

[283] Riaz A., Lagnika C., Luo H., Nie M., Dai Z., Liu C., Abdin M., Hashim M.M., Li D., Song J. (2020). Effect of Chinese chives (*Allium tuberosum*) addition to carboxymethyl cellulose based food packaging films. Carbohydr. Polym..

[284] Kaur R., Kaur L. (2021). Encapsulated natural antimicrobials: A promising way to reduce microbial growth in different food systems. Food Control.

[285] Delshadi R., Bahrami A., Assadpour E., Williams L., Jafari S.M. (2021). Nano/microencapsulated natural antimicrobials to control the spoilage microorganisms and pathogens in different food products. Food Control.

[286] Dima C., Assadpour E., Dima S., Jafari S.M. (2020). Bioactive-loaded nanocarriers for functional foods: From designing to bioavailability. Curr. Opin. Food Sci..

[287] Yousefi M., Ehsani A., Jafari S.M. (2019). Lipid-based nano delivery of antimicrobials to control food-borne bacteria. Adv. Colloid Interface Sci..

[288] Burgain J., Gaiani C., Linder M., Scher J. (2011). Encapsulation of probiotic living cells: From laboratory scale to industrial applications. J. Food Eng..

[289] Jafari S.M., Assadpoor E., He Y., Bhandari B. (2008). Encapsulation Efficiency of Food Flavours and Oils during Spray Drying. Dry. Technol..

[290] Mahdavi S.A., Jafari S.M., Ghorbani M., Assadpoor E. (2014). Spray-Drying Microencapsulation of Anthocyanins by Natural Biopolymers: A Review. Dry. Technol..

[291] Jafari S.M., Jafari S.M. (2017). 1—An overview of nanoencapsulation techniques and their classification. Nanoencapsulation Technologies for the Food and Nutraceutical Industries.

[292] Aloui H., Khwaldia K., Licciardello F., Mazzaglia A., Muratore G., Hamdi M., Restuccia C. (2014). Efficacy of the combined application of chitosan and Locust Bean Gum with different citrus essential oils to control postharvest spoilage caused by Aspergillus flavus in dates. Int. J. Food Microbiol..

[293] Huang Q., Given P. (2009). 1952- Micro/Nano Encapsulation of Active Food Ingredients.

[294] Akhavan Mahdavi S., Jafari S.M., Assadpoor E., Dehnad D. (2016). Microencapsulation optimization of natural anthocyanins with maltodextrin, gum Arabic and gelatin. Int. J. Biol. Macromol..

[295] Suganya V., Anuradha V. (2017). Microencapsulation and Nanoencapsulation: A Review. Int. J. Pharm. Clin. Res..

[296] Anastasi E., Riviere G., Teste B., ANSES-French Agency for Food, Environmental and Occupational Health & Safety, France (2019). Nanomaterials in Food-Prioritisation & Assessment. EFSA J..

[297] Chen L., Remondetto G.E., Subirade M. (2006). Food protein-based materials as nutraceutical delivery systems. Trends Food Sci. Technol..

[298] Joye I.J., McClements D.J. (2013). Production of nanoparticles by anti-solvent precipitation for use in food systems. Trends Food Sci. Technol..

[299] Khare A.R., Vasisht N., Gaonkar A.G., Vasisht N., Khare A.R., Sobel R. (2014). Chapter 14—Nanoencapsulation in the Food Industry: Technology of the Future. Microencapsulation in the Food Industry.

[300] Zanetti M., Carniel T.K., Dalcanton F., dos Anjos R.S., Gracher Riella H., de Araújo P.H.H., de Oliveira D., Antônio Fiori M. (2018). Use of encapsulated natural compounds as antimicrobial additives in food packaging: A brief review. Trends Food Sci. Technol..

[301] Heckert Bastos L.P., Vicente J., Corrêa dos Santos C.H., Geraldo de Carvalho M., Garcia-Rojas E.E. (2020). Encapsulation of black pepper (*Piper nigrum* L.) essential oil with gelatin and sodium alginate by complex coacervation. Food Hydrocoll..

[302] Misra S., Trinavee K., Gunda N.S.K., Mitra S.K. (2020). Encapsulation with an interfacial liquid layer: Robust and efficient liquid-liquid wrapping. J. Colloid Interface Sci..

[303] LaCoste A., Schaich K.M., Zumbrunnen D., Yam K.L. (2005). Advancing controlled release packaging through smart blending. Packag. Technol. Sci..

[304] Buonocore G.G., Nobile M.A.D., Panizza A., Bove S., Battaglia G., Nicolais L. (2003). Modeling the Lysozyme Release Kinetics from Antimicrobial Films Intended for Food Packaging Applications. J. Food Sci..

[305] Chen X., Chen M., Xu C., Yam K.L. (2019). Critical review of controlled release packaging to improve food safety and quality. Crit. Rev. Food Sci. Nutr..

[306] Yam K.L., Zhu X. (2012). Controlled release food and beverage packaging. Emerging Food Packaging Technologies.

[307] Uz M., Altınkaya S.A. (2011). Development of mono and multilayer antimicrobial food packaging materials for controlled release of potassium sorbate. LWT-Food Sci. Technol..

[308] Beikzadeh S., Akbarinejad A., Swift S., Perera J., Kilmartin P.A., Travas-Sejdic J. (2020). Cellulose acetate electrospun nanofibers encapsulating Lemon Myrtle essential oil as active agent with potent and sustainable antimicrobial activity. React. Funct. Polym..

[309] Bi F., Qin Y., Chen D., Kan J., Liu J. (2021). Development of active packaging films based on chitosan and nano-encapsulated luteolin. Int. J. Biol. Macromol..

[310] Lei K., Wang X., Li X., Wang L. (2019). The innovative fabrication and applications of carvacrol nanoemulsions, carboxymethyl chitosan microgels and their composite films. Colloids Surf. B Biointerfaces.

[311] Alves V.L.C.D., Rico B.P.M., Cruz R.M.S., Vicente A.A., Khmelinskii I., Vieira M.C. (2018). Preparation and characterization of a chitosan film with grape seed extract-carvacrol microcapsules and its effect on the shelf-life of refrigerated Salmon (Salmo salar). LWT.

[312] Martiñon M.E., Moreira R.G., Castell-Perez M.E., Gomes C. (2014). Development of a multilayered antimicrobial edible coating for shelf-life extension of fresh-cut cantaloupe (*Cucumis melo* L.) stored at 4 °C. LWT-Food Sci. Technol..

[313] Sadeghi K., Seo J. (2021). Photografting Coating: An Innovative Approach to “Non-Migratory” Active Packaging. Adv. Funct. Mater..

[314] Gundewadi G., Rudra S.G., Sarkar D.J., Singh D. (2018). Nanoemulsion based alginate organic coating for shelf life extension of okra. Food Packag. Shelf Life.

[315] Lee J.Y., Garcia C.V., Shin G.H., Kim J.T. (2019). Antibacterial and antioxidant properties of hydroxypropyl methylcellulose-based active composite films incorporating oregano essential oil nanoemulsions. LWT.

[316] Mohsenabadi N., Rajaei A., Tabatabaei M., Mohsenifar A. (2018). Physical and antimicrobial properties of starch-carboxy methyl cellulose film containing rosemary essential oils encapsulated in chitosan nanogel. Int. J. Biol. Macromol..

[317] Nazari M., Majdi H., Milani M., Abbaspour-Ravasjani S., Hamishehkar H., Lim L.-T. (2019). Cinnamon nanophytosomes embedded electrospun nanofiber: Its effects on microbial quality and shelf-life of shrimp as a novel packaging. Food Packag. Shelf Life.

[318] Hossain F., Follett P., Salmieri S., Vu K.D., Fraschini C., Lacroix M. (2019). Antifungal activities of combined treatments of irradiation and essential oils (EOs) encapsulated chitosan nanocomposite films in in vitro and in situ conditions. Int. J. Food Microbiol..

[319] Aydogdu A., Yildiz E., Aydogdu Y., Sumnu G., Sahin S., Ayhan Z. (2019). Enhancing oxidative stability of walnuts by using gallic acid loaded lentil flour based electrospun nanofibers as active packaging material. Food Hydrocoll..

[320] Hardy A., Benford D., Halldorsson T., Jeger M.J., Knutsen H.K., More S., Naegeli H., Noteborn H., Ockleford C., EFSA Scientific Committee (2018). Guidance on risk assessment of the application of nanoscience and nanotechnologies in the food and feed chain: Part 1, human and animal health. EFSA J..

